# Misvaluation and technological acquisitions: An empirical study and mechanism analysis

**DOI:** 10.1371/journal.pone.0313848

**Published:** 2024-11-14

**Authors:** Jinwei Zhou, Qizheng Gao, Qi Luo

**Affiliations:** 1 Economics and Management School, Wuhan University, Wuhan, Hubei, China; 2 Business School of Hunan Normal University, Changsha, Hunan, China; Tianjin University, CHINA

## Abstract

This paper investigates the influence of industry-level overvaluation on technological acquisitions, utilizing merger and acquisition (M&A) data from Chinese listed firms spanning 2007 to 2022. Our analysis confirms that industry-level overvaluation promotes technological acquisitions, a finding that remains significant even after several robustness checks. Compared to acquirers with lower financial constraints, those with higher financial constraints rely more on industry-level overvaluation to drive technological acquisitions. Furthermore, the study indicates that technological acquisitions initiated by industry-level overvaluation tend to enhance the innovative output of the acquirer. By exploring the relationship between industry-level overvaluation and technological acquisitions, this research extends the literature on misvaluation-driven M&A. Additionally, this study provides new evidence and perspectives on the impact of capital markets on the real economy, and contributes to the healthy development of capital markets and M&A markets in emerging countries.

## 1 Introduction

One of the primary functions of capital markets is to support the development of the real economy. Research by Shleifer and Vishny [1] and Rhodes-Kropf and Viswanathan [2] demonstrates that merger and acquisition (M&A) activities are significantly influenced by the pricing efficiency of capital markets. Recent research has also provided evidence that overvaluation promotes M&As [3]. In recent years, with the rapid development of digital technologies [4–6], companies face new opportunities and challenges, and innovation activities are becoming increasingly complex. In this context, technological acquisitions are growing in importance as an important means of acquiring new skills and technological knowledge. Technological acquisitions can absorb the technological knowledge of target companies, enhancing the technological expertise and capabilities of the acquirer [7]. This study expands the research scope of misvaluation-driven M&As by exploring the relationship between misvaluation and technological acquisitions.

Recent empirical evidence on misvaluation-driven acquisitions primarily comes from market-level [8] or firm-level [9] analyses, with limited evidence at the industry level. The Chinese stock market exhibits characteristics of rapid correction of short-term misjudgments of firm value [10], and the overvaluation of individual acquirers can be offset by the undervaluation of non-acquiring firms within the same industry [11]. These factors may constrain the overall impact of firm-level misvaluation on acquisition activities. In contrast, industry-level overvaluation suggests growth potential in the industry [3], potentially exerting a more persistent influence on acquisition activities. Technological acquisitions play a crucial role in enhancing a firm’s innovative capabilities [7, 12]. However, existing research has not yet fully explored the specific mechanisms by which industry-level misvaluation influences technological acquisitions. Further examination into whether industry-level misvaluation has a dominant influence on technological acquisition decisions would expand our understanding of the role of misvaluation in technological acquisition decision-making.

There is currently no consensus in the literature on whether misvaluation-driven M&As can achieve synergies. Some studies suggest that M&As driven by misvaluation fail to produce positive synergies [13], while others find that M&As conducted by companies with overvalued stocks can generate higher synergistic benefits [14]. This divergence highlights the necessity for in-depth research on this issue, especially in the field of technological acquisitions. Technological acquisitions play a crucial role in enhancing corporate innovation capabilities, and innovation output can be seen as an important manifestation of M&A synergies. However, existing research has not fully explored how misvaluation-driven technological acquisitions affect innovation output. In the context of China’s economic transition towards high-quality development and increasingly frequent technological acquisitions, this study aims to fill this research gap. Specifically, we examine the impact of industry-level misvaluation on technological acquisitions and explore how these acquisitions subsequently affect acquirers’ innovation output.

Existing research on the antecedents of technological acquisitions primarily focuses on internal factors, including organizational size [15], innovation efficiency [16], organizational structure [17], and managerial characteristics [18]. While these studies identify significant internal determinants, research on external influences remains relatively limited. Investigations into external factors are generally confined to M&As, with insufficient attention to technological acquisitions specifically. Recent empirical studies in the Chinese context have explored the impact of factors such as political corruption [19] and Buddhist beliefs [20] on acquisition activities. Notably, despite the critical role of technological acquisitions in enhancing firms’ innovative capabilities [7, 12], academic discussion on this topic remains limited.

Recent literature has explored the influence of non-traditional external factors such as Buddhist beliefs, but in reality, these factors may have minimal impact on technological acquisition decisions. In contrast, considering the close connection between the stock market and merger waves [1, 21], examining the impact of misvaluation on technological acquisitions would be more meaningful. As one of the most attractive emerging markets in Asia in recent years, China presents a case worthy of attention. China’s capital market is relatively new and still faces challenges such as an imperfect institutional framework and low market maturity [22]. Unlike many other markets dominated by institutional investors, China’s market has a relatively high proportion of retail investors [23]. The tendency for irrational behavior among retail investors may intensify market volatility, thus affecting the stability of listed companies’ stock prices [24]. Given these characteristics of the Chinese market, studying the impact of misvaluation on technological acquisitions in China is of significant importance.

In China, a transitional economy, firms often face internal resource and capability constraints [25]. These constraints lead to path dependence in technological research and development. Consequently, it becomes difficult for these firms to achieve breakthrough innovations. Open innovation provides firms with a viable path to overcome these difficulties, enabling them to acquire core capabilities and strategic resources externally [26]. However, existing literature predominantly focuses on the concept of open innovation [27], with limited exploration of the influencing factors driving firms’ adoption of this strategy. Among the various methods of open innovation, technological acquisition is considered one of the most crucial [28]. It not only brings complementary innovative resources to firms but also reduces organizational inertia, enhancing innovation output and R&D efficiency [29]. Therefore, an in-depth study of the antecedents influencing technological acquisitions not only helps to understand corporate technological acquisition behavior but also reveals key factors shaping firms’ engagement in open innovation strategies.

In summary, although existing research has extensively explored the impact of misvaluation on M&As, several limitations persist. First, insufficient attention is given to technological acquisitions as a key strategy for firms to achieve open innovation. Second, in examining the antecedents of technological acquisitions, studies have predominantly focused on internal factors while neglecting the role of external capital markets. Finally, extant research has primarily concentrated on market-level and firm-level misvaluation, with a relatively scant exploration of industry-level misvaluation. Given the differences between China’s stock market and mature markets, it is necessary to investigate the impact of misvaluation on technological acquisitions based on the Chinese market context. However, our understanding of how industry-level misvaluation drives technological acquisitions and subsequently influences acquirers’ innovation output remains limited.

Given these considerations, our study aims to examine the relationship between industry-level misvaluation and technological acquisitions in the Chinese stock market, thereby contributing to a relatively unexplored domain in the extant literature. Specifically, we analyze the impact of industry-level misvaluation on technological acquisitions by Chinese listed firms. Our results show that industry-level overvaluation significantly promotes technological acquisitions. Gorbenko and Malenko [30] point out that the relationship between corporate financial constraints and M&As is not yet fully understood, particularly in the Chinese context. Our research demonstrates that firms with greater financial constraints are more likely to rely on industry-level overvaluation to drive technological acquisitions compared to those with fewer financial constraints. This finding complements Gorbenko and Malenko’s call for further research on this topic.

Further, our research findings indicate that technological acquisitions driven by industry-level overvaluation can enhance the acquiring firm’s innovation output. The synergistic effects are manifested not only in financial performance but also in improved innovation output. Previous studies have shown that M&As driven by misvaluation may bring short-term financial gains to acquirers but are detrimental to long-term economic efficiency and value creation [1]. In contrast, our study finds that technological acquisitions motivated by industry-level overvaluation can lead to increased innovation output. Moreover, most existing research has examined the impact of misvaluation on corporate innovation from a closed innovation perspective, including equity financing channels [31] and catering channels [32]. Our research extends the scope to open innovation by investigating how misvaluation influences technological acquisitions. Given that technological acquisitions are a key pathway for implementing open innovation, our study demonstrates how industry-level overvaluation influences these acquisitions. This finding provides new insights into the influencing factors of corporate engagement in open innovation. However, as this study focuses solely on the Chinese market, future research should consider expanding the scope to include other countries and regions.

The rest of the paper is organized as follows: Section 2 provides the literature review. Section 3 presents the theoretical analysis and research hypotheses. Section 4 details the research methodology and empirical design. Section 5 discusses the empirical results and analyses. Section 6 offers further analyses. Section 7 presents the discussion. Section 8 concludes.

## 2 Literature review

### 2.1 Technological acquisitions

Technological acquisition, as a crucial strategic choice for firms to obtain external innovative capabilities, has garnered significant attention from academia in recent years. However, extant research on technological acquisitions still exhibits limitations and controversies regarding both antecedents and subsequent performance. Concerning the antecedents of technological acquisitions, scholars have primarily focused on internal firm factors, such as company size, innovation efficiency, and managerial characteristics. Andersson and Xiao [15] demonstrate that large firms typically enhance their innovation potential by acquiring technology-intensive smaller firms. Bena and Li [16] find that firms with high innovation efficiency tend to acquire those with low innovation efficiency. Furthermore, Lin et al. [18] indicate that CEOs with innovation practice experience are more likely to initiate technological acquisitions. However, these studies emphasize internal corporate factors while neglecting the external market environment, particularly the potential impact of misvaluation on technological acquisition decisions.

In addition, academic consensus has not yet been reached regarding the impact of technological acquisitions on firms’ innovation performance. Scholars holding positive views offer various perspectives on this matter. For instance, Sun et al. [12] argue that technological acquisitions can expand the acquirer’s knowledge base. Jin et al. [33] find that such acquisitions help acquiring firms address competitive pressures. Furthermore, Lee and Lee [34] explore the impact of target and acquirer characteristics on innovation performance, noting that closer geographical proximity and higher technological similarity between target and acquiring firms can increase joint patent applications post-acquisition. Conversely, studies with negative perspectives, such as Graebner et al. [35], caution that the complexity of technological acquisitions often leads to disappointing post-acquisition outcomes. These findings suggest that our understanding of the relationship between technological acquisitions and innovation performance may be limited. Technological acquisition decisions do not exist in isolation from the external environment but are significantly influenced by capital market factors. This interdependence highlights the necessity of examining corporate technological acquisition behavior and its subsequent innovation performance in conjunction with misvaluation factors.

### 2.2 Misvaluation and M&A

The relationship between market misvaluation and M&As has been extensively studied in the literature. Shleifer and Vishny [1] and Rhodes-Kropf and Viswanathan [2] provide theoretical explanations for how market valuation influences M&A activities. Rhodes-Kropf et al. [21] validate these theories empirically, offering direct support for the impact of misvaluation on M&A activities. More recently, Huang et al. [3] find that market overvaluation promotes cross-border M&A activities. Nie and Yen [8] elucidate that during periods of high investor sentiment, which often signal market overvaluation, overconfident CEOs are more likely to engage in M&As. Lai and Pu [9] reveal the influence of target-specific misvaluation on private equity acquirers’ decision-making. These studies have provided valuable insights into how misvaluation affects M&A activities. However, it is noteworthy that existing research primarily focuses on the impact of market-level and firm-level misvaluation on M&As, while evidence on the influence of industry-level misvaluation on technological acquisitions remains scarce.

Academic consensus regarding the economic consequences of market-driven M&As has not been reached. Adra and Barbopoulos [14] find that acquisitions by firms with overvalued stocks may yield higher synergistic gains. Research based on the Chinese market indicates that non-state-owned enterprises experience positive market reactions during M&A announcements, while state-owned enterprises demonstrate more significant advantages in long-term performance improvement post-acquisition [36]. However, Vagenas-Nanos [37] posits that overvalued acquirers fail to create synergies through M&As. Zhang et al. [13] find that overvaluation-driven acquisitions may only improve performance in the current year without achieving long-term synergies. Although these studies offer important insights into the relationship between market valuation and M&As, research limitations persist. While previous studies have discussed the impact of misvaluation-driven M&As on firms’ market and accounting performance, research on their influence on innovation output remains insufficient. Moreover, the existing literature has not adequately explored the dominant role of misvaluation at different levels (e.g., firm-level, industry-level) in influencing technological acquisition decisions, nor how such misvaluation-driven technological acquisitions affect acquirers’ innovation output. This study aims to fill this potential research gap by examining whether industry-level overvaluation has a greater impact on technological acquisitions compared to firm-level overvaluation, while also exploring the effect of such acquisitions on innovation output.

## 3 Theoretical analysis and hypothesis development

### 3.1 Misvaluation and technological acquisitions

Shleifer and Vishny [1] introduce the Stock Market Driven Acquisitions (SMDA) theory, which suggests a positive correlation between overvalued stock prices and increased M&A activity. Misvaluation sources are multifaceted, with both firm-level and industry-level misvaluations escalating market misvaluation [2]. However, a clear difference exists between firm-level and industry-level misvaluations in driving technological acquisitions. Industry-level overvaluation reflects market optimism regarding the entire industry and its future growth potential [3], and the market may exhibit a greater propensity to support technological acquisitions by firms within that industry. According to the information asymmetry theory [38], the internal management of a company has more accurate knowledge of the company’s true value than external investors. The Chinese capital market is highly volatile, and the information disclosure mechanism is inadequate [39]. In this context, outside investors may believe that the industry’s long-term growth potential can offset the high risk associated with technological acquisitions. Industry-level overvaluation may reduce the difficulties in reaching acquisition agreements, thereby facilitating the occurrence of technological acquisitions.

In the Chinese market, prudent lending decisions by creditors inhibit firms’ acquisition behavior [40]. When a firm’s access to capital is restricted, the lack of financial support affects its ability to conduct acquisitions, especially technological acquisitions. In such cases, managers become more cautious in utilizing the firm’s available capital, preferring to allocate limited funds to production activities and daily operations [41]. According to the market timing hypothesis in capital structure theory, firms exhibit a propensity to raise capital under favorable market conditions, leveraging overvaluation to reduce their cost of capital [42]. The stock market can alleviate the financial pressure on firms and enable them to obtain the necessary capital to support innovative strategic decisions. In China’s transitional economy, the most concentrated and profitable industries tend to experience overvaluation in the stock market [43]. Within this market context, industry-level overvaluation is likely to increase the probability of firms engaging in technological acquisitions.

In contrast to industry-level overvaluation, the impact of firm-level overvaluation on technological acquisition behavior may be relatively limited. Short-term misjudgments of firm value in the Chinese stock market tend to be rapidly corrected [10]. Furthermore, the overvaluation of an individual acquirer’s stock is likely to be offset by the undervaluation of other firms within the same industry [11]. Consequently, firm-level overvaluation exhibits greater instability, which weakens its role in driving technological acquisitions. These characteristics of short-term overvaluation may also elicit concerns from target firms regarding the risks associated with technological acquisitions. In such circumstances, only acquiring firms possess a detailed understanding of undervaluation risks. This disparity exacerbates information asymmetry, leading target firms to scrutinize more cautiously the proposals from overvalued acquirers, particularly in the high-risk domain of technological acquisitions. This suggests that industry-level overvaluation may play a more significant role in driving technological acquisitions compared to firm-level overvaluation. Based on this analysis, the following hypotheses are proposed:

H1: Industry-level overvaluation promotes technological acquisitions.

### 3.2 Misvaluation, financial constraints and technological acquisitions

China’s capital market, a relative latecomer compared to established markets, still suffers from imperfect institutional frameworks, low market maturity, and pervasive information asymmetry [22]. These factors contribute to high external financing costs and limited financing channels, preventing firms from investing at optimal levels. Financing constraints are particularly prevalent among Chinese firms, significantly hindering their innovative activities [44]. Such constraints are recognized as major obstacles to achieving breakthrough innovations [45]. Successful firms’ innovation activities typically rely on adequate financial support, with external financing being a crucial source [46]. However, firms often encounter difficulties in securing bank loans due to insufficient collateral [47]. When the cost of innovation capital increases, firms’ innovation activities are consequently inhibited [48]. Although the disclosure of R&D activities can enhance corporate innovation [49], firms are typically hesitant to disclose such information due to concerns about competitors gaining access to their trade secrets. This hesitation, in turn, exacerbates the challenges associated with securing external financing.

Technological acquisitions are a key strategy for firms to enhance innovation by directly obtaining technological resources and knowledge [50]. However, technological acquisitions are typically highly opaque, making it challenging for acquirers to accurately assess the value of the target firm’s resources, a challenge that is pronounced among Chinese firms [51]. Information asymmetry renders technological acquisitions characterized by high uncertainty, leading to difficulties in realizing post-acquisition synergies and potentially hindering the development of other R&D projects due to the reallocation of funds and manpower, thereby affecting overall innovation capability [52]. Additionally, the reallocation of intra-firm innovation following technological acquisitions is influenced by factors such as the firm’s life cycle and product market competition, which further exacerbate uncertainty [53]. Moreover, technological acquisitions typically involve highly specialized assets and uncertain future returns, complicating the assessment of the value and potential risks of the acquisition [35]. These risks and uncertainties make technological acquisitions more dependent on the stock market, especially when firms are subject to significant financing constraints.

While technological acquisitions can improve firms’ innovative capabilities and foster their development, they also require a large amount of capital investment [54]. In a highly constrained financing environment, firms rely more heavily on equity financing, and overvalued stock prices can significantly reduce the cost of equity financing. Moreover, the impact of overvalued stock prices on a firm’s equity financing is amplified as the level of financing constraints increases [55]. When stock prices are overvalued, using shares to pay for M&A transactions becomes less costly than using cash. The acquirer can either partially or wholly use the shares for payment, mitigating the risks inherent in cash-reliant technological acquisitions. As a result, industry-level overvaluation can alleviate financial constraints for acquirers, facilitating technological acquisition. Drawing from this analysis, the paper proposes the following hypothesis:

H2: Firms with high financial constraints depend more on industry-level overvaluation to facilitate technological acquisitions than less financially constrained firms.

## 4 Research design

### 4.1 Variables construction

#### 4.1.1 Dependent variables: Technological acquisition

**Fig 1:**
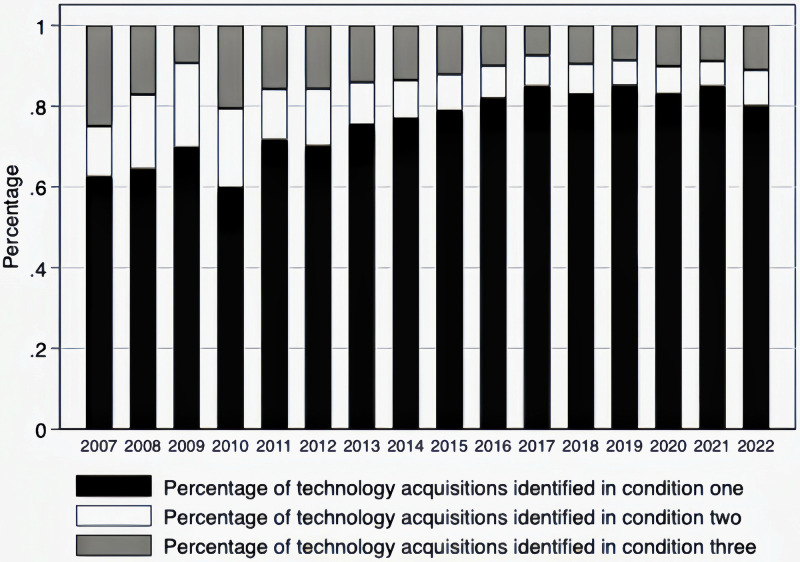
Distribution of technological acquisitions sample identifications.

This paper adopts Ahuja and Katila [7] definition of technological acquisitions and proposes that a firm-initiated M&A qualifies as a technological acquisition under one of three conditions. Firstly, the acquisition is acknowledged by the acquirer in the M&A announcement as being motivated primarily by technology considerations. Secondly, the acquisition target has secured patents within the five years before the M&A. Finally, the M&A transaction occurs within an industry characterized by emerging or advanced technologies. Contrary views from some scholars highlight that merely having acquired patents does not necessarily equate to a technological acquisition, especially if the patents are of negligible value. Additionally, to address the debate over patent valuation, Fig 1 presents a stacked bar chart that confirms a significant portion of our sample of technological acquisitions fulfills the first condition.

#### 4.1.2 Independent variables: Industry-level misvaluation

Prior research predominantly uses the price-to-book (P/B) and price-to-earnings (P/E) ratios as indicators of market misvaluation [56, 57]. However, these ratios are susceptible to misvaluation and investment opportunities, making it difficult to isolate these two effects. Rhodes-Kropf et al. [21] addressed this issue by applying yearly industry-specific regressions to the market-to-book ratio (M/B), categorizing the results into firm-level misvaluation, industry-level misvaluation, and growth opportunity. Given its widespread academic recognition, we draw upon the methodology of Rhodes-Kropf et al. [21], which deconstructs the market-to-book ratio (M/B) into two distinct components: market misvaluation and fundamental long-term valuation.

$$\begin{array}{c} Market/Book\equiv Market/Value\times Value/Book \end{array}$$ (1)

*Market to Value* represents market misvaluation, while *Value to Book* is an indicator of the company’s growth prospects. A *Market to Value* ratio of 1 indicates that the stock is reasonably priced to accurately reflect the company’s inherent growth. Rhodes-Kropf et al. [21] argue that Eq (1) can be disaggregated into Eq (2), which provides a more detailed analysis of these components.

$$\begin{array}{c} m_{it}-b_{it}=\underset{firm\;}{\underset{︸}{m_{it}-v(\theta_{it};a_{jt})}}+\underset{industry\;}{\underset{︸}{v(\theta_{it};a_{jt})-v(\theta_{it};a_{j})}}+\underset{growth}{\underset{︸}{v(\theta_{it};a_{j})-b_{it}}} \end{array}$$ (2)

In this context, *m*_*it*_ and *b*_*it*_ refer to the natural logarithms of total market value of equity and total assets, respectively. *v*(*θ*_*it*_;*a*_*jt*_)represents the intrinsic value of firm *i* within a specific year *t* and industry *j*, while *v*(*θ*_*it*_; *a*_*j*_)corresponds to the fundamental value attributed to firm *i* within industry *j*.

This study concentrates on the effects of industry-level misvaluation on technological acquisitions. The formula for calculating industry-level misvaluation is as follows:

$$\begin{array}{c} Industry\;Misvaluation=v(\theta_{it};a_{jt})-v(\theta_{it};a_{j}) \end{array}$$ (3)

*Industry Misvaluation* is approaching 0 implying more efficient long-term market pricing. Conversely, values greater than 0 suggest an overvaluation at the industry level, whereas values less than 0 indicate an undervaluation within the industry.

We adopt the approach of Rhodes-Kropf et al. [21] by initially regressing the total market value of equity against the natural logarithm of total assets, the natural logarithm of net income, and the leverage ratio. As shown in Eq (4):

$$\begin{array}{c} m_{it}=a_{0t}+a_{1t}b_{it}+a_{2t}{NI}_{it}+a_{3t}{Lev}_{it}+\varepsilon \end{array}$$ (4)

Eq (4) is regressed on year and industry. We follow the Securities and Exchange Commission (SEC) Industry Classification Standard, 2012 edition, manufacturing is categorized into broad secondary groups, whereas other industries are classified into primary sectors.

We regress Eq (4) by year and industry, and calculate the regression coefficients for each year by industry.

$$\begin{array}{c} v(\theta_{it};a_{jt})={\hat{a}}_{0t}+{\hat{a}}_{1t}b_{it}+{\hat{a}}_{2t}{NI}_{it}+{\hat{a}}_{3t}{Lev}_{it} \end{array}$$ (5)

Further, we average the model regression coefficients by industry for each year, and calculate ${\bar{a}}_{j}=1/T\sum a_{tj}$. Then we have Eq (6):

$$\begin{array}{c} v(\theta_{it};a_{j})={\bar{a}}_{0}+{\bar{a}}_{1}b_{it}+{\bar{a}}_{2}{NI}_{it}+{\bar{a}}_{3}{Lev}_{it} \end{array}$$ (6)

In summary, the industry-level misvaluation of firm *i* in period *t* is computed using Eq (3) through Eq (6).

#### 4.1.3 Measurement of financial constraint indicators

Numerous scholars have employed the *KZ index* [58], the *SA index* [59], and the *WW index* [60] as metrics to ascertain the extent of financing constraints confronting firms. In this study, we initially apply the *SA index* to gauge firms’ financing constraints, given its exclusion of financing variables with endogenous properties. To ensure our findings are robust across different indices, we conduct additional robustness analyses using both the *KZ index* and the *WW index*.

We refer to Hadlock and Pierce [59] for the calculation of the *SA index*:

$$\begin{array}{c} SA\;index=-0.737\times Size+0.043\times Size^{2}-0.040\times Age \end{array}$$ (7)

where *Size* denotes the natural logarithm of the firm’s total asset and *Age* denotes the firm’s operating year.

In the robustness of the mechanism test, we draw on Ding et al. [58] to calculate the *KZ index*:

$$\begin{array}{c} \begin{array}{c} KZ\;index_{i,t}=-1.002\times CF_{i,t}/Assets_{i,t-1}+0.283\times Q_{i,t}+3.139\times Debt_{i,t}/Assets_{i,t}\\ -39.368\times DIV_{i,t}/Assets_{i,t-1}-1.315\times Cash_{i,t}/Assets_{i,t-1} \end{array} \end{array}$$ (8)

In this formulation, *CF* signifies cash flow, *Assets* represents total assets, *Q* denotes Tobin’s Q, *Debt* encapsulates total debt (computed as the aggregate of short-term and long-term debt), *DIV* refers to dividends, and *Cash* indicates cash and cash equivalents. A higher value of the *KZ index* suggests that the firm is encumbered with more pronounced financing constraints.

We also used the *WW index* [60] as an alternative measure of financing constraints, calculated as follows:

$$\begin{array}{cc} WW\;index= & -0.091\times Cashflow/Asset-0.062\times DIVPOS+0.021\times LTDT/Asset\\ & -0.044\times Size+0.102\times ISG-0.035\times SG \end{array}$$ (9)

*DIVPOS* is a binary variable, taking the value of 1 if the company distributes a cash dividend and 0 if it does not. *LTDT* represents the long-term debt of the company. *ISG* denotes the sales growth of the company within its three-digit industry classification, while *SG* stands for the company’s overall sales growth. Corporate financing constraints intensify as the *WW index* increases.

#### 4.1.4 Innovation outputs

Drawing on Cloodt et al. [61], we quantify post-acquisition innovation output through the number of patents granted. Specifically, we use the natural logarithm of the total number of patents granted (plus one) two years after the technological acquisition, as well as the natural logarithm of the number of patents granted for inventions (plus one), measured two years after technological acquisition.

### 4.2 Data sources

This study conducts an empirical analysis of A-share listed companies in China over the period 2007 to 2022, and the selection criteria for the sample are as follows: (i) listed companies as the acquirer; (ii) inclusion of transactions where the M&A was completed; (iii) exclusion of financial institutions; and (iv) exclusion of companies with missing essential variables. The resulting sample consists of 11,834 observations. we applied winsorization to all continuous variables at the 1st and 99th percentiles to mitigate the effects of outliers. We sourced the financial data for this analysis from the China Stock Market & Accounting Research (CSMAR) database.

The CSMAR database, which stands for China Stock Market & Accounting Research Database, is a research-oriented and precise database intended to serve academic institutions and financial organizations for research and quantitative investment analysis purposes. The CSMAR database follows the classification standards of well-known databases such as CRSP and COMPUSTAT, and it is adapted to the specific conditions of the domestic financial market as well as the research habits of academic institutions and organizations. It is divided into 11 series, including stocks, companies, funds, bonds, derivatives, economics, industries, and overseas markets, encompassing more than 80 databases.

### 4.3 Empirical models

This paper examines the impact of industry-level misvaluation on technological acquisitions using the following regression model:

$$\begin{array}{c} TADecision_{i,t}=a_{0}+a_{1}Industry\;Misvaluation_{i,t-1}+a_{i}Controls_{i,t-1}+\omega_{i}+\mu_{t}+\varepsilon_{it} \end{array}$$ (10)

The dependent variable, *TADecision*_*i*,*t*_, represents the technological acquisition decisions of firm *i* in year *t* and comprises three dimensions. These dimensions include whether technological acquisitions occurred (*TAdummy*), measured as a 0–1 variable; the frequency of technological acquisitions (*TAcount*), recorded as a count variable; and the scale of technological acquisitions (*TAratio*), expressed as a ratio. Given the numerical characteristics of the explanatory variables, we employed Logit, Poisson, and OLS regression models to regress Eq (10) respectively. *Industry Misvaluation*_*i*,*t*−1_ denotes industry-level misvaluation for firm *i* in year *t* − 1, while *Controls*_*i*,*t*−1_ denotes a set of control variables that could affect the technological acquisitions of firms. Additionally, *ω*_*i*_ represents firm fixed effect, *μ*_*t*_ is year fixed effect, and standard errors are clustered at the industry level. To mitigate concerns of endogeneity, such as reverse causation, we introduce single-epoch lags for all independent variables in our analysis. The definitions of the variables used in the empirical analysis are provided in Table 1.

**Table 1:** List of variable definitions.

Variable Names	Variable Meanings	Variable Descriptions
TA Motivation	Technological acquisitions motivation	*TAdummy* denotes the dummy variable for the firm instigating a technological acquisition in the given year, taking a value of 1 for the occurrence of a technological acquisition and 0 otherwise
		*TAcount* signifies the quantity of technological acquisitions instigated by the firm within the year
		*TAratio* is a measure representing the total value of technological acquisition deals initiated by list firms during the year as a percentage of the previous year’s total assets
*Industry Misvaluation*	Industry-level misvaluation	Industry-level misvaluation from M/B decomposition
*Long-run Performance*	Company growth	Company growth from M/B decomposition
*Firm Misvaluation*	Firm-level misvaluation	Firm-level misvaluation from M/B decomposition
*RD*	R&D investment	R&D expenditure /operating revenue
*Size*	Company size	Natural logarithm of total assets
*OCF*	Cash flows	Net cash flows from operating activities / total assets at end of period
*Yretwd*	Annual individual stock returns	Annual individual stock returns considering reinvestment of cash dividends
*PPE*	Proportion of fixed assets	Net fixed assets/total assets
*Board*	Board size	Natural logarithm of the number of board members
*Dual*	Dual position holding	*Dual* indicates whether the chairman and the CEO are the same person; 0: No; 1: Yes
*IND*	Proportion of independent directors	The ratio of the number of independent directors to the total board size
*Shares Balance*	Shares balance ratio	The combined shareholding of the 2nd to 5th largest shareholders divided by the shareholding of the largest shareholder
*Insinvestor*	Institutional investor shareholding ratio	The proportion of shares held by institutional investors in a listed company
*Attendance*	Annual shareholders’ meeting attendance rate	The attendance rate at the annual shareholders’ meeting
*Board Meetings*	Number of board meetings	The number of board meetings held
*Payment*	Payment method	1 for cash, 2 for equity, and 3 for a combination of equity and cash, and 0 for other payment methods
*Target Type*	Target types	1 for equity, and 2 for a combination of equity and assets, and 0 for other target types

## 5 Empirical results and analysis

### 5.1 Descriptive statistics

Table 2 presents the descriptive statistics for the primary variables. Within the sample period, 46.21% of firms initiated at least one technological acquisition. The highest number of technological acquisitions in a single year stands at four. This finding highlights that technological acquisition has become a prevalent and critical innovation strategy for companies, necessitating an analysis of this behavior. The average *Industry Misvaluation* is 0.1247, suggesting a modest overvaluation of stock prices at the industry level, and there is no significant systematic deviation between stock and book value. The standard deviation of 0.3258 in *Industry Misvaluation* indicates a substantial variance in stock price overvaluation across industries. Additionally, in S1 Table, we provide the descriptive statistics of the sample’s net income and leverage ratio.

**Table 2:** Summary statistics.

Variables	Obs	Mean	Std. Dev.	Min	Median	Max	p1	p99
*TAdummy*	11834	0.4621	0.4986	0.0000	0.0000	1.0000	0.0000	1.0000
*TAcount*	11834	0.7794	1.0773	0.0000	0.0000	4.0000	0.0000	4.0000
*TAratio*	11834	0.0326	0.0861	0.0000	0.0000	0.8751	0.0000	0.4435
*Industry Misvaluation*	11834	0.1247	0.3258	-1.1059	0.0617	1.3710	-0.6220	0.9330
*Long-run Performance*	11834	0.4282	0.5091	-0.8652	0.3720	1.5204	-0.8644	1.5204
*Firm Misvaluation*	11834	0.0023	0.3789	-1.4111	0.0093	1.9778	-0.8022	1.1338
*RD*	11834	4.7180	3.8700	0.0300	4.4300	23.9500	0.0300	23.3100
*Size*	11834	21.9757	1.0721	19.8050	21.9225	25.1623	19.8050	25.1167
*OCF*	11834	0.0462	0.0620	-0.1231	0.0402	0.2095	-0.1231	0.2094
*Yretwd*	11834	0.2503	0.6440	-0.6646	0.1931	2.9298	-0.6646	2.9298
*PPE*	11834	0.1964	0.1368	0.0095	0.1656	0.5898	0.0097	0.5890
*Board*	11834	2.1206	0.1867	1.6094	2.1972	2.6391	1.6094	2.6391
*Dual*	11834	0.3111	0.4424	0.0000	0.0000	1.0000	0.0000	1.0000
*IND*	11834	0.3756	0.0538	0.0000	0.3636	1.0000	0.3333	0.5714
*Shares Balance*	11834	0.7852	0.6157	0.0096	0.6774	4.0000	0.0329	2.8544
*Insinvestor*	11834	0.4090	0.2366	0.0000	0.4115	1.0114	0.0030	0.9040
*Attendance*	11834	0.4983	0.1385	0.0006	0.5029	1.0000	0.1507	0.8251
*Board Meetings*	11834	10.2516	3.7830	2.0000	10.0000	55.0000	4.0000	22.0000
*Payment*	11834	0.9088	0.5096	0.0000	1.0000	3.0000	0.0000	3.0000
*Target Type*	11834	0.7812	0.4181	0.0000	1.0000	2.0000	0.0000	1.0000

### 5.2 Correlation analysis

The correlation analysis in Table 3 reveals that the key dependent variable, *TAdummy*, exhibits significant correlations with several other variables. Specifically, *TAdummy* shows significant positive correlations with *Industry Misvaluation*, *Size*, among other variables. The significant positive correlation between *Industry Misvaluation* and *TAdummy* suggests that higher industry misvaluation is associated with an increased probability of a firm undertaking a technological acquisition. Similarly, *Size* exhibits a significant positive correlation with *TAdummy*, indicating that larger companies are more likely to engage in technological acquisitions. This finding is consistent with previous research, which also suggests that larger firms are more inclined to undertake technological acquisitions [15]. This study includes three dependent variables, for which we have conducted separate correlation analyses. Given that the focus of this paper is to examine whether industry-level misvaluation promotes the occurrence of technological acquisitions, we primarily present and analyze the correlation between *TAdummy* and other variables. The correlation analyses for *TAcount* and *TAratio* are provided in the S3 and S4 Tables.

**Table 3:** Correlation analysis.

	*TAdummy*	*Industry Misvaluation*	*Long-run Performance*	*Firm Misvaluation*	*RD*	*Size*	*OCF*	*Yretwd*	*PPE*	*Board*	*Dual*	*IND*	*Shares Balance*	*Insinvestor*	*Attendance*	*Board Meetings*	*Payment*	*Target Type*
*TAdummy*	1																	
*Industry Misvaluation*	0.101***	1																
*Long-run Performance*	0.079***	0.022**	1															
*Firm Misvaluation*	0.017*	0.006	0.001	1														
*RD*	0.153***	0.041***	0.267***	0.115***	1													
*Size*	0.065***	0.082***	-0.671***	0.039***	-0.164***	1												
*OCF*	0.024***	0.030***	0.118***	0.047***	-0.007	0.066***	1											
*Yretwd*	-0.081***	0.313***	0.024***	0.226***	0.002	-0.006	0.079***	1										
*PPE*	-0.064***	-0.037***	-0.129***	-0.039***	-0.197***	0.083***	0.220***	0.046***	1									
*Board*	-0.081***	-0.059***	-0.193***	-0.010	-0.093***	0.206***	0.027***	0.030***	0.106***	1								
*Dual*	0.076***	0.031***	0.155***	0.067***	0.105***	-0.146***	-0.009	-0.017*	-0.101***	-0.147***	1							
*IND*	0.022**	0.031***	0.035***	0.017*	0.051***	-0.024***	-0.003	-0.027***	-0.045***	-0.424***	0.103***	1						
*Shares Balance*	0.054***	0.019**	0.120***	0.046***	0.143***	-0.088***	-0.025***	-0.023***	-0.090***	0.001	0.042***	0	1					
*Insinvestor*	-0.104***	-0.028***	-0.249***	0.117***	-0.154***	0.369***	0.122***	0.066***	0.125***	0.214***	-0.161***	-0.084***	-0.216***	1				
*Attendance*	0.024***	-0.003	0.148***	0.074***	-0.0140	-0.058***	0.106***	-0.006	-0.065***	-0.009	0.070***	0.006	-0.010	0.201***	1			
*Board Meetings*	0.016*	0.082***	-0.174***	0.070***	-0.032***	0.245***	-0.097***	0.069***	-0.048***	-0.040***	-0.017**	0.046***	0.002	0.030***	-0.103***	1		
*Payment*	0.097***	0.093***	0.027***	0.011	0.060***	0.046***	0.004	-0.072***	-0.039***	-0.095***	0.073***	0.056***	0.058***	-0.090***	-0.007	0.054***	1	
*Target Type*	0.170***	0.116***	0.026***	0.016*	0.083***	0.071***	0.032***	-0.132***	-0.061***	-0.134***	0.088***	0.067***	0.102***	-0.128***	-0.030***	0.019**	0.577***	1

Additionally, we observed correlations among the control variables. For example, there is a significant negative correlation between Firm Size (*Size*) and *Long-run Performance*, while *Size* shows a significant positive correlation with Institutional Investor Shareholding Ratio (*Insinvestor*). To address potential multicollinearity issues, we calculated the Variance Inflation Factor (VIF) for all explanatory variables. If the VIF of an explanatory variable exceeds 10, it may indicate a multicollinearity problem. The VIF results, presented in S2 Table, show that all VIF values are below the threshold of 10, indicating that multicollinearity is not a serious concern in this analysis. Thus, our regression model is not affected by multicollinearity, ensuring the reliability of our research results.

### 5.3 Fundamental regression results

Table 4 presents the regression results for the industry-level misvaluation and technological acquisitions. For the benchmark regressions, this study adopts a progressive regression approach. Columns (1), (3), and (5) include only firm-level and year-specific fixed effects, while columns (2), (4), and (6) introduce additional control variables relating to the firm’s financial performance and governance characteristics.

**Table 4:** Baseline regression results.

	(1)	(2)	(3)	(4)	(5)	(6)
	Logit		Poisson		OLS	
Variables	*TAdummy*	*TAdummy*	*TAcount*	*TAcount*	*TAratio*	*TAratio*
*Industry Misvaluation*	0.5656***	0.4839***	0.2241***	0.2009***	0.0206***	0.0187***
	(4.0945)	(3.2839)	(3.2814)	(2.7053)	(4.9558)	(4.0548)
*Long-run Performance*		0.3957***		0.1688***		0.0166***
		(3.8203)		(3.5477)		(4.0031)
*Firm Misvaluation*		0.2116**		0.1192***		0.0041
		(2.0525)		(2.6750)		(1.4416)
*RD*		-0.0058		-0.0148		-0.0006
		(-0.4291)		(-1.6231)		(-1.6774)
*Size*		0.5764***		0.2010***		0.0022
		(7.2932)		(5.1963)		(0.8352)
*OCF*		0.9369*		0.4338		0.0290
		(1.7392)		(1.3786)		(1.4655)
*Yretwd*		-0.2759***		-0.1169***		-0.0033*
		(-4.3634)		(-5.5923)		(-1.6908)
*PPE*		0.3121		0.1506		0.0183
		(0.8355)		(0.4992)		(1.5005)
*Board*		-0.0436		0.1200		-0.0117
		(-0.1718)		(0.8080)		(-1.1564)
*Dual*		0.0680		0.0446		0.0024
		(0.7296)		(1.0881)		(0.6614)
*IND*		-0.4271		-0.2591		-0.0182
		(-0.5316)		(-0.8480)		(-0.5054)
*Shares Balance*		0.0182		-0.0367		0.0038
		(0.1948)		(-0.8398)		(1.1113)
*Insinvestor*		-0.5798*		-0.0163		0.0006
		(-1.8675)		(-0.1127)		(0.0703)
*Attendance*		1.6814***		0.5672***		0.0233**
		(5.5261)		(4.5510)		(2.2651)
*Board Meetings*		-0.0019		0.0039		0.0018***
		(-0.1854)		(1.3144)		(4.1705)
*Payment*		-0.2109***		-0.0951***		0.0122***
		(-3.0925)		(-2.9320)		(3.0954)
*Target Type*		0.2554**		0.1421**		0.0002
		(2.3882)		(2.4920)		(0.0583)
Constant			0.1678***	-4.7742***	0.0300***	-0.0402
			(14.9698)	(-4.4325)	(54.6826)	(-0.6072)
Year fixed effect	Yes	Yes	Yes	Yes	Yes	Yes
Firm fixed effect	Yes	Yes	Yes	Yes	Yes	Yes
Observations	7760	7760	9051	9051	11834	11834
Adj. R^2^					0.104	0.115
Pseudo R^2^	0.122	0.142	0.137	0.141		

Note: *TAdummy* denotes the dummy variable for the firm instigating a technological acquisition in the given year, taking a value of 1 for the occurrence of a technological acquisition and 0 otherwise. *TAcount* signifies the quantity of technological acquisitions instigated by the firm within the year. *TAratio* is a measure representing the total value of technological acquisition deals initiated by list firms during the year as a percentage of the previous year’s total assets. The fixed-effects logistic regression analysis for panel data, which excludes variables uniformly zero or one, resulted in a decreased sample size, yielding 7,760 observations. In contrast, the fixed-effects Poisson regression analysis for panel data excludes only those variables that are consistently zero, leading to a marginally larger sample size of 9,051. Statistical z-values are reported in parentheses beneath columns (1) to (4), whilst t-values are detailed for columns (5) and (6). In the tables that follow, the symbols ***, **, and * represent statistical significance levels of 1%, 5%, and 10%, respectively.

In the fixed-effects Logit model, without including control variables but controlling for firm and year fixed effects, the regression coefficient for *Industry Misvaluation* is 0.5656. When control variables are introduced while still controlling for firm and year fixed effects, the regression coefficient for *Industry Misvaluation* is 0.4839. The coefficient of 0.5656 indicates that when *Industry Misvaluation* increases by 0.01 units, the odds of engaging in a technological acquisition (*TAdummy* = 1) relative to not engaging in a technological acquisition (*TAdummy* = 0) increase by approximately 0.5672%. This percentage change is calculated as [exp(0.5656 × 0.01) − 1] × 100%.

The regression analysis results depicted in Table 4 indicate a statistically significant and positive correlation between industry-level overvaluation and technological acquisitions, thus supporting H1. The reduction in the coefficient of *Industry Misvaluation* after introducing control variables is because these control variables capture some of the effects originally attributed to *Industry Misvaluation*, thereby reducing omitted variable bias. This adjustment makes the coefficients more reflective of their true effects, enhancing the accuracy and reliability of the model estimates.

### 5.4 Robustness checks

#### 5.4.1 Excluding competition theory explanations

The firm life cycle theory suggests that a firm’s investment opportunities and valuations grow with its maturation. Additionally, as industry competition evolved towards oligopolistic structures, companies were compelled to seek ways to surmount technological constraints and enhance their innovative capabilities, often pursuing technological acquisitions. Therefore, the uplift in firm valuations and the increase in technological acquisitions are intrinsic hallmarks of firm life cycle laws. To control for potential confounding effects associated with the firm life cycle, both the squared and linear terms of firm age are incorporated as control variables in the regression analysis. The results presented in Table 5, Panel A, columns (1) through (3), indicate that *Industry Misvaluation* remains positive at the 1% significance level, thereby mitigating the influence of the firm life cycle on the observed relationship.

**Table 5:** Robustness check—Excluding competing hypotheses and PSM-DID results.

	Panel A			Panel B			Panel C		
	(1)	(2)	(3)	(1)	(2)	(3)	(1)	(2)	(3)
	Logit	Poisson	OLS	Logit	Poisson	OLS	Logit	Poisson	OLS
Variables	*TAdummy*	*TAcount*	*TAratio*	*TAdummy*	*TAcount*	*TAratio*	*TAdummy*	*TAcount*	*TAratio*
*Industry Misvaluation*	0.5176***	0.1945***	0.0186***	0.4755***	0.1900***	0.0186***			
	(3.4879)	(2.6768)	(3.8546)	(3.2189)	(2.6505)	(4.1353)			
*PostTreat*							-0.4709*	-0.1765*	-0.0139***
							(-1.7743)	(-1.6558)	(-2.8218)
*Long-run Performance*	0.3886***	0.1734***	0.0167***	0.3996***	0.1684***	0.0166***	0.4182	0.0284	0.0063
	(3.7436)	(3.6707)	(4.0716)	(3.8521)	(3.4145)	(3.9378)	(1.6122)	(0.3006)	(0.8999)
*Firm Misvaluation*	0.2155**	0.1131**	0.0039	0.2097**	0.1183***	0.0040	0.1252	0.0751	-0.0005
	(2.0760)	(2.5143)	(1.3675)	(2.0332)	(2.6454)	(1.4130)	(0.5710)	(0.8864)	(-0.0928)
*RD*	-0.0043	-0.0145	-0.0006*	-0.0068	-0.0156*	-0.0007*	0.0639*	0.0071	0.0003
	(-0.3161)	(-1.6154)	(-1.7125)	(-0.5008)	(-1.6758)	(-1.8629)	(1.7856)	(0.6445)	(0.3499)
*Size*	0.6152***	0.2001***	0.0019	0.5828***	0.2170***	0.0023	0.7573***	0.1550*	-0.0049
	(7.5900)	(5.1385)	(0.7675)	(7.1598)	(5.6576)	(0.8433)	(3.7143)	(1.8514)	(-0.5929)
*OCF*	0.9814*	0.4417	0.0291	0.9470*	0.4325	0.0306	-1.2427	-0.3942	0.0219
	(1.8179)	(1.4073)	(1.4767)	(1.7566)	(1.3434)	(1.5299)	(-1.0100)	(-0.6535)	(0.3578)
*Yretwd*	-0.2796***	-0.1154***	-0.0033*	-0.2724***	-0.1145***	-0.0031	-0.2384*	-0.0759	-0.0020
	(-4.4102)	(-5.5931)	(-1.7089)	(-4.2973)	(-5.7467)	(-1.5911)	(-1.7949)	(-1.3989)	(-0.6433)
*PPE*	0.4791	0.1467	0.0175	0.3077	0.1355	0.0187	-0.3351	-0.3421	-0.0093
	(1.2631)	(0.4885)	(1.4187)	(0.8231)	(0.4476)	(1.5210)	(-0.3606)	(-0.6921)	(-0.2979)
*Board*	-0.0795	0.1276	-0.0114	-0.0421	0.1128	-0.0115	0.9057	0.5808*	-0.0062
	(-0.3119)	(0.8724)	(-1.1250)	(-0.1660)	(0.7595)	(-1.1350)	(1.3540)	(1.6503)	(-0.2950)
*Dual*	0.0603	0.0459	0.0025	0.0669	0.0442	0.0023	-0.0252	-0.0750	-0.0022
	(0.6467)	(1.1274)	(0.6836)	(0.7173)	(1.0775)	(0.6421)	(-0.1265)	(-0.7047)	(-0.2972)
*IND*	-0.4377	-0.2741	-0.0186	-0.4417	-0.2650	-0.0199	0.3667	1.6466*	-0.0240
	(-0.5440)	(-0.8912)	(-0.5190)	(-0.5492)	(-0.8787)	(-0.5540)	(0.1912)	(1.7006)	(-0.4467)
*Shares Balance*	-0.0040	-0.0333	0.0041	0.0146	-0.0398	0.0036	-0.1081	0.0161	0.0113**
	(-0.0427)	(-0.7604)	(1.2229)	(0.1553)	(-0.9480)	(1.0561)	(-0.5029)	(0.2560)	(2.0310)
*Insinvestor*	-0.6092**	-0.0127	0.0009	-0.5759*	-0.0209	0.0015	-1.6841**	-0.2835	0.0010
	(-1.9605)	(-0.0879)	(0.1096)	(-1.8544)	(-0.1458)	(0.1766)	(-2.2707)	(-0.8774)	(0.0571)
*Attendance*	1.4129***	0.5961***	0.0255**	1.6839***	0.5657***	0.0230**	1.0521	-0.0116	-0.0194
	(4.3594)	(4.3379)	(2.2112)	(5.5089)	(4.5379)	(2.2387)	(1.5575)	(-0.0347)	(-0.8804)
*Board Meetings*	-0.0032	0.0038	0.0018***	-0.0025	0.0040	0.0017***	-0.0008	0.0071	0.0019**
	(-0.3138)	(1.2841)	(4.2126)	(-0.2516)	(1.1870)	(4.0276)	(-0.0331)	(0.9381)	(2.3656)
*Payment*	-0.2187***	-0.0957***	0.0122***	-0.2111***	-0.0949***	0.0122***	-0.1359	-0.0148	0.0159**
	(-3.2014)	(-2.9879)	(3.1137)	(-3.0927)	(-2.9566)	(3.0908)	(-0.8058)	(-0.2301)	(2.3404)
*Target Type*	0.2714**	0.1402**	0.0001	0.2561**	0.1442**	0.0001	-0.1272	-0.0592	-0.0065
	(2.5289)	(2.5025)	(0.0421)	(2.3945)	(2.5575)	(0.0512)	(-0.5362)	(-0.7199)	(-0.6213)
*Age*	0.6002**	0.2396**	0.0095**						
	(2.3123)	(2.4970)	(2.1096)						
*Age* ^2^	0.0023***	-0.0000	-0.0000						
	(2.7532)	(-0.1555)	(-0.3237)						
*Overhead rate*				0.4720	0.8885***	0.0183			
				(0.5814)	(3.1257)	(1.1712)			
*Total Asset Turnover*				-0.0427	0.0727	-0.0063			
				(-0.2686)	(0.7642)	(-1.5847)			
*Other Accounts Receivable*				0.5424	-0.3735	0.0529			
				(0.3577)	(-0.3793)	(1.5205)			
Constant		-7.0153***	-0.1308*		-5.2206***	-0.0407		-4.6877**	0.1377
		(-4.4699)	(-1.7041)		(-4.8261)	(-0.5823)		(-2.1040)	(0.8636)
Year fixed effect	Yes	Yes	Yes	Yes	Yes	Yes	Yes	Yes	Yes
Firm fixed effect	Yes	Yes	Yes	Yes	Yes	Yes	Yes	Yes	Yes
Observations	7760	9051	11834	7760	9051	11834	1507	2258	3225
Adj. *R*^2^			0.115			0.116			0.192
Pseudo *R*^2^	0.144	0.141		0.142	0.141		0.144	0.160	

Note: *TAdummy* denotes the dummy variable for the firm instigating a technological acquisition in the given year, taking a value of 1 for the occurrence of a technological acquisition and 0 otherwise. *TAcount* signifies the quantity of technological acquisitions instigated by the firm within the year. *TAratio* is a measure representing the total value of technological acquisition deals initiated by list firms during the year as a percentage of the previous year’s total assets. In Panel A, Panel B, and Panel C, column (3) encloses the t-values in parentheses. Additionally, parentheses enclose the z-values for the remaining regressions.

Excluding the life cycle theory, the coefficient for *Industry Misvaluation* is 0.5176. This indicates a positive correlation between *Industry Misvaluation* and *TAdummy*. The coefficient of 0.5176 indicates that when *Industry Misvaluation* increases by 0.01 units, the odds of engaging in a technological acquisition relative to not engaging increase by approximately 0.5189%. This percentage change is calculated as [exp(0.5176 × 0.01) − 1] × 100%.

Furthermore, technological acquisitions are more likely to prioritize market response over value creation, as managers or majority shareholders may engage in such deals to serve their interests. To accommodate potential interference from agency costs, variables are included in the regression related to the first and the second types of agency costs. The findings in Table 5, Panel B, demonstrate significant positives between *Industry Misvaluation* and technological acquisitions at the 1% significance levels, reinforcing the initial conclusion that industry-level overvaluation drives technological acquisitions.

When excluding the agency theory, the coefficient for *Industry Misvaluation* changes slightly to 0.4755. This still reflects a positive correlation between *Industry Misvaluation* and *TAdummy*. The coefficient of 0.4755 suggests that when *Industry Misvaluation* rises by 0.01 units, the odds of pursuing a technological acquisition compared to not pursuing one increase by approximately 0.4766%. This percentage change is computed as [exp(0.4755 × 0.01) − 1] × 100%. The conclusion remains valid even after controlling for agency costs, highlighting the pivotal role of industry-level overvaluation in driving technological acquisitions.

#### 5.4.2 PSM-DID

The theoretical analysis of this study posits that industry-level overvaluation promotes technological acquisitions. However, as the pricing efficiency of capital markets improves, the incentive for companies to initiate technological acquisitions when stock prices are overvalued is weakened. Short selling and margin trading (SSMT) can effectively improve stock pricing efficiency [62].

Short selling and margin trading (SSMT) refers to the practice where investors can borrow funds from brokerage firms to purchase stocks (margin trading) or borrow stocks to sell them (short selling). This mechanism allows investors to buy stocks through margin trading when they expect stock prices to rise or sell stocks through short selling when they expect prices to fall. This enables the leverage effect of funds and helps achieve the purpose of hedging risks. China began piloting SSMT in 2008 and officially expanded it nationwide in 2010. The policy aims to stimulate market activity, enhance liquidity, and provide investors with more tools for investment and risk management. By increasing market trading activity and participation, SSMT helps to quickly incorporate information into stock prices, thereby improving the efficiency of price discovery in the market. SSMT reforms are independent of firms’ technological acquisition decisions and include securities selected based on market capitalization and liquidity, rather than technological activity. Therefore, SSMT is considered exogenous and naturally delineates the control and treatment groups.

PSM is an efficient statistical method that estimates the propensity score for each subject under given covariates by considering multiple relevant covariates. This method effectively balances differences in observed characteristics between the treatment and control groups, thus improving matching quality, reducing selection bias, and improving the interpretability of the results. Compared to simple matching, PSM reduces selection bias by balancing covariates, increasing the reliability of statistical analysis and helping researchers approximate the conditions of a randomized experiment, thus allowing for more accurate estimation of treatment effects. This approach is particularly important in observational studies because it helps achieve conditional independence between the treatment and control groups.

**Fig 2:**
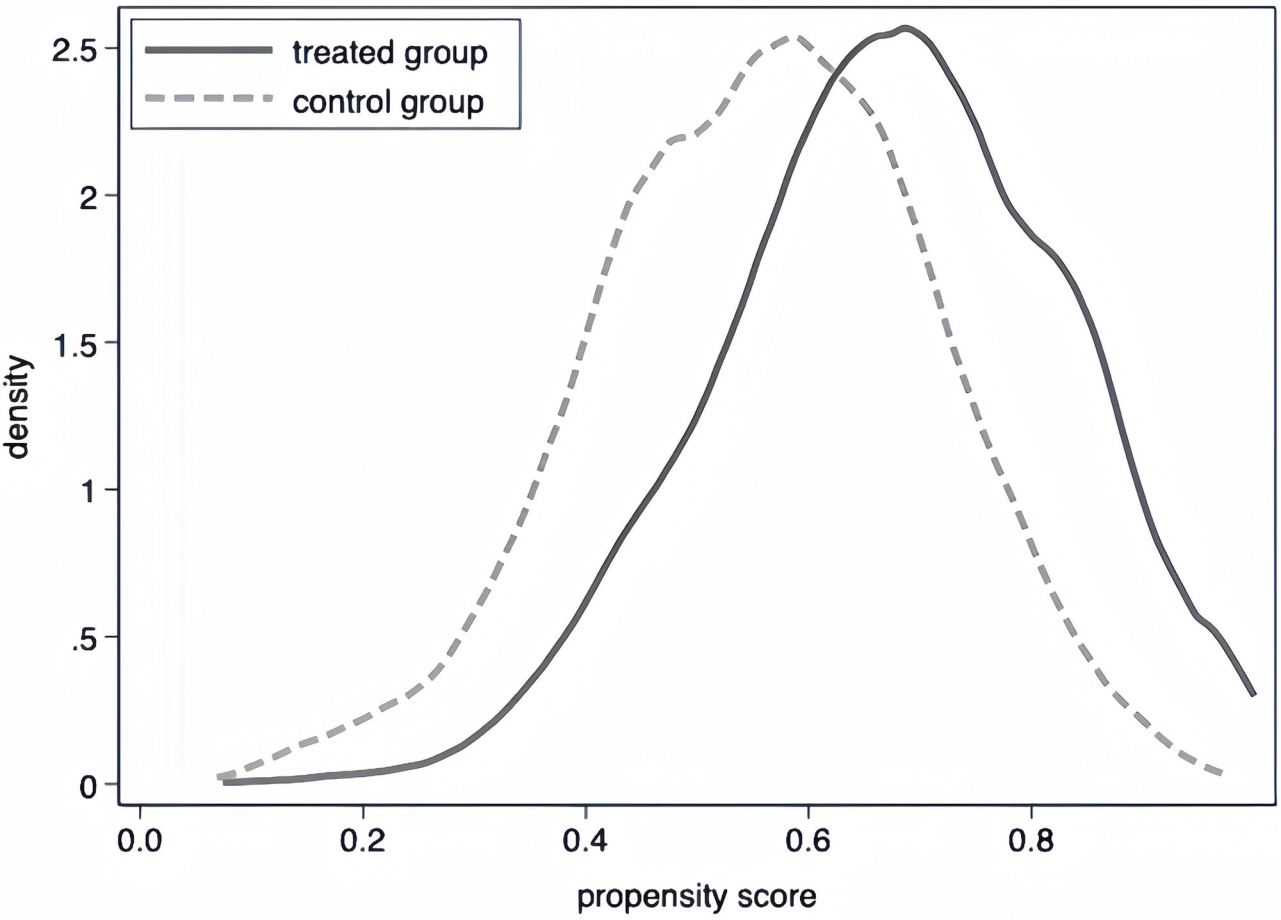
Kernel density before matching.

**Fig 3:**
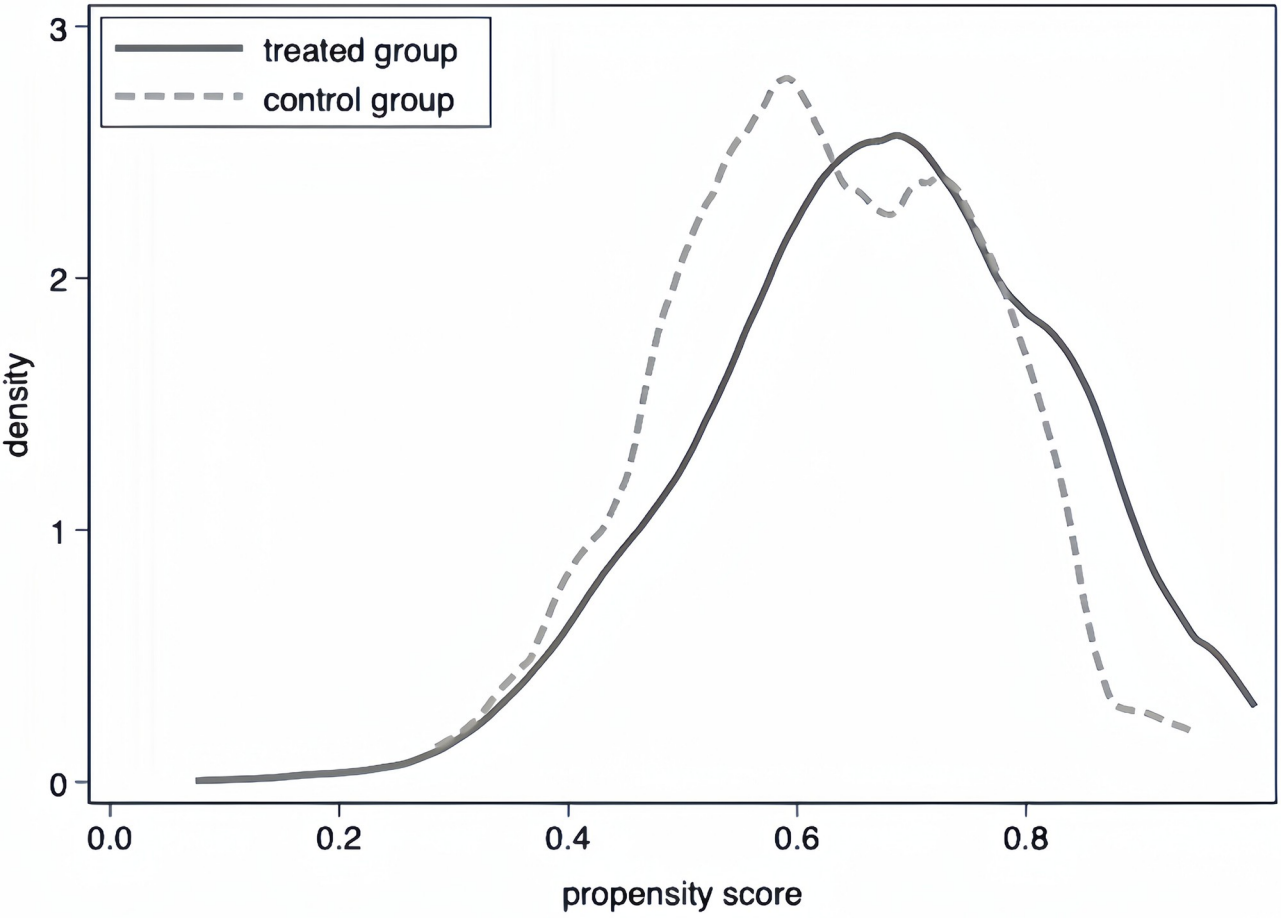
Kernel density after matching.

For constructing the PSM sample, we implement a 1:1 nearest-neighbor matching method on a year-to-year basis without replacement, identifying a control group for SSMT subjects. We draw on studies by Xu et al. [63], which used kernel density maps to demonstrate the quality of the match. The kernel densities of the treated and untreated groups before and after matching are depicted in Figs 2 and 3 showing negligible differences between these groups after matching. We also present the results of the PSM balance test in S5 Table, where matching significantly reduced the t-values of the covariates, thereby eliminating the apparent differences between the treatment and control groups.

**Fig 4:**
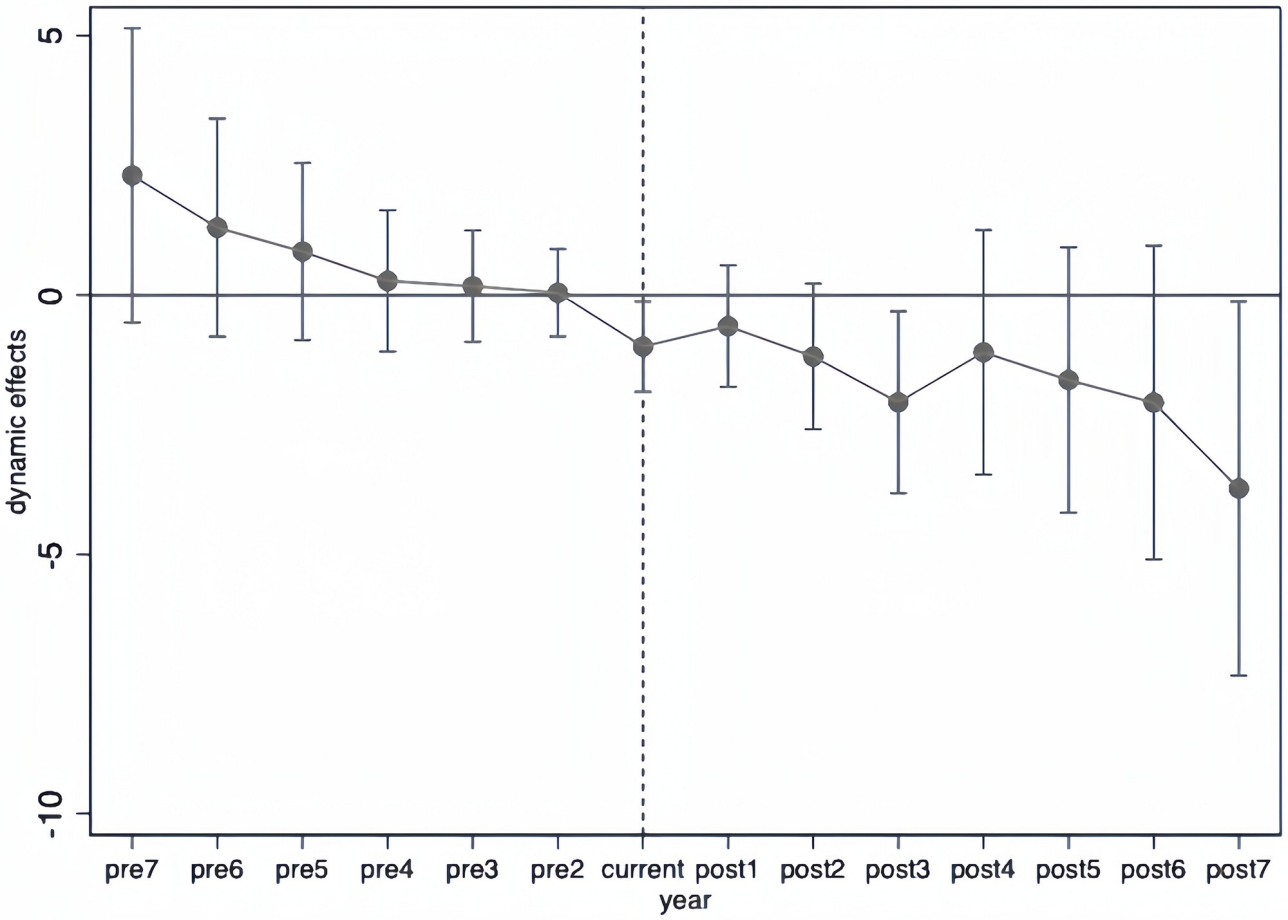
Parallel trend test.

In reality, policies are essentially nonrandomized experiments or quasi-natural experiments. Therefore, the DID method used for policy effect evaluation inevitably suffers from self-selection bias. Using PSM can match each treated sample with a specific control sample, making the quasi-natural experiment approximate randomness. Consequently, PSM can make the DID estimation results closer to the true causal effect and improve estimation efficiency. To ensure the validity of our approach, we conduct a parallel trends test between the treatment and control groups. The results of this parallel trend test are presented in Fig 4. We employ the PSM-DID method to examine the impact of industry-level misvaluation on technological acquisitions, with the regression model displayed in Eq (11):

$$\begin{array}{c} TADecision_{i,t}=a_{0}+a_{1}Post_{i,t}\times Treat_{i,t}+a_{i}Controls_{i,t-1}+\omega_{i}+\mu_{t}+\varepsilon_{it} \end{array}$$ (11)

We initially define the time variable, denoted as *Time*, representing the number of years relative to the SSMT event year for each company. For example, if a company underwent SSMT in 2013, *Time* would be set to -4 for that company in 2009. Additionally, we create a time difference variable, labeled *Post*. *Post* is assigned a value of 0 when a firm’s Time is less than 0 in a given year. Otherwise, it is assigned a value of 1. Subsequently, we establish a second difference variable denoted as *Treat*, is established to differentiate firms directly involved in SSMT (treatment group, *Treat* = 1) from their corresponding matched (control group, *Treat* = 0). Due to the covariance issue, *Post* and *Treat* are not introduced separately in the model. The interaction terms’ regression coefficients, presented in Table 5, Panel C, are significantly negative. This negative relationship suggests that listed firms undergoing SSMT exhibit a decreased level of overvaluation and a significantly lower propensity to initiate technological acquisitions relative to the control group. These empirical findings support the theoretical analysis proposed in this study, underscoring the significant impact of industry-level overvaluation on technological acquisitions.

#### 5.4.3 Alternative explanatory variables and sample intervals

Some scholars have suggested that to address the potential bias in regression results caused by excluding companies with negative net profits, the explanatory variable should be replaced with the market-to-book ratios. Additionally, to address concerns about analyzing acquisitions from 2007 in 2022, we adjusted the sample period to include only acquisitions from the most recent five years. The results of these tests are presented in Table 6. As shown in Panel A, *MB* remains significant at the 1% level.

**Table 6:** Robustness check—Alternative explanatory variables and sample intervals.

	Panel A			Panel B		
	(1)	(2)	(3)	(1)	(2)	(3)
	Logit	Poisson	OLS	Logit	Poisson	OLS
Variables	*TAdummy*	*TAcount*	*TAratio*	*TAdummy*	*TAcount*	*TAratio*
*MB*	0.4839***	0.2009***	0.0187***			
	(3.2839)	(2.7053)	(4.0548)			
*Industry Misvaluation*				0.9877**	0.2480**	0.0195*
				(2.3013)	(2.1255)	(1.7168)
*Long-run Performance*	0.3957***	0.1688***	0.0166***	0.7691***	0.2832***	0.0329***
	(3.8203)	(3.5477)	(4.0031)	(3.1590)	(2.6643)	(4.2003)
*Firm Misvaluation*	-0.2722	-0.0818	-0.0146**	0.7850***	0.1726	0.0004
	(-1.5941)	(-0.8343)	(-2.4876)	(3.4431)	(1.5886)	(0.0582)
*RD*	-0.0058	-0.0148	-0.0006	-0.0177	-0.0217	-0.0012
	(-0.4291)	(-1.6231)	(-1.6774)	(-0.5970)	(-1.1449)	(-0.6757)
*Size*	0.5764***	0.2010***	0.0022	0.6270**	0.1881**	0.0011
	(7.2932)	(5.1963)	(0.8352)	(2.5094)	(2.0038)	(0.1158)
*OCF*	0.9369*	0.4338	0.0290	1.0302	0.3034	0.0187
	(1.7392)	(1.3786)	(1.4655)	(0.9239)	(0.4801)	(0.4901)
*Yretwd*	-0.2759***	-0.1169***	-0.0033*	-0.3680***	-0.1413**	0.0026
	(-4.3634)	(-5.5923)	(-1.6908)	(-2.6159)	(-2.3213)	(0.9112)
*PPE*	0.3121	0.1506	0.0183	1.2262	0.8885*	0.0832**
	(0.8355)	(0.4992)	(1.5005)	(1.2541)	(1.8213)	(2.1792)
*Board*	-0.0436	0.1200	-0.0117	-0.4549	0.1777	-0.0103
	(-0.1718)	(0.8080)	(-1.1564)	(-0.8114)	(0.7810)	(-0.5817)
*Dual*	0.0680	0.0446	0.0024	-0.2357	0.0008	-0.0052
	(0.7296)	(1.0881)	(0.6614)	(-1.1097)	(0.0092)	(-0.6159)
*IND*	-0.4271	-0.2591	-0.0182	-1.9019	-0.6355**	0.0082
	(-0.5316)	(-0.8480)	(-0.5054)	(-1.5387)	(-2.1589)	(0.1207)
*Shares Balance*	0.0182	-0.0367	0.0038	2.4002***	1.0368***	0.0788**
	(0.1948)	(-0.8398)	(1.1113)	(3.1194)	(3.8674)	(2.3721)
*Insinvestor*	-0.5798*	-0.0163	0.0006	-0.0119	0.0022	0.0026***
	(-1.8675)	(-0.1127)	(0.0703)	(-0.5575)	(0.2197)	(4.6458)
*Attendance*	1.6814***	0.5672***	0.0233**	-0.5996**	-0.2150**	-0.0042
	(5.5261)	(4.5510)	(2.2651)	(-2.3958)	(-2.3893)	(-0.3529)
*Board Meetings*	-0.0019	0.0039	0.0018***	0.1133	0.0965	0.0157
	(-0.1854)	(1.3144)	(4.1705)	(0.3910)	(0.6007)	(1.3796)
*Payment*	-0.2109***	-0.0951***	0.0122***	0.9877**	0.2480**	0.0195*
	(-3.0925)	(-2.9320)	(3.0954)	(2.3013)	(2.1255)	(1.7168)
*Target Type*	0.2554**	0.1421**	0.0002	0.7691***	0.2832***	0.0329***
	(2.3882)	(2.4920)	(0.0583)	(3.1590)	(2.6643)	(4.2003)
Constant		-4.7742***	-0.0402		-4.6965**	-0.0629
		(-4.4325)	(-0.6072)		(-2.0399)	(-0.3121)
Observations	7760	9051	11834	1888	3109	3649
Adj. R^2^			0.115			0.083
Pseudo R^2^	0.142	0.141		0.093	0.130	

Note: *TAdummy* denotes the dummy variable for the firm instigating a technological acquisition in the given year, taking a value of 1 for the occurrence of a technological acquisition and 0 otherwise. *TAcount* signifies the quantity of technological acquisitions instigated by the firm within the year. *TAratio* is a measure representing the total value of technological acquisition deals initiated by list firms during the year as a percentage of the previous year’s total assets. In Panel A, Panel B, and Panel C, column (3) encloses the t-values in parentheses. Additionally, parentheses enclose the z-values for the remaining regressions.

In Panel B, *Industry Misvaluation* is significantly positive at the 5% and 10% levels. Under the scenario of changing the sample interval, the coefficient for *Industry Misvaluation* is 0.9877, which is significantly positive at the 5% level. The estimated coefficient of 0.9877 implies that a 0.01-unit increment in *Industry Misvaluation* is associated with an approximately 0.9926% increase in the odds of initiating a technological acquisition versus not doing so. This percentage is derived using the formula [exp(0.9877 × 0.01) − 1] × 100%. This finding further corroborates the crucial role that industry misvaluation plays in explaining technological acquisition behavior.

Furthermore, we conduct supplementary robustness checks to solidify the validity of benchmark regression findings. These robustness checks involved using alternative regression models and excluding observations from anomalous periods such as financial crises, stock market crashes, and the COVID-19 pandemic. Detailed regression results can be found in S6 Table.

### 5.5 Mechanism test

Initially, this study draws upon Hadlock and Pierce [59], which utilizes the *SA index* to measure a firm’s financing constraints. Firms categorized within the (*High FC*) subsample demonstrate *SA index* values that meet or exceed the annual median. In contrast, the (*Low FC*) subsample is derived through an inverse process. Table 7 displays the regression results for the mechanism test, revealing a significantly positive coefficient for *Industry Misvaluation* at the 1% level within the (*High FC*) subsample—a pattern not mirrored in the (*Low FC*) subsample.

**Table 7:** Mechanism test.

	(1)	(2)	(3)	(4)	(5)	(6)
	*High FC*	*Low FC*	*High FC*	*Low FC*	*High FC*	*Low FC*
	Logit		Poisson		OLS	
Variables	*TAdummy*	*TAdummy*	*TAcount*	*TAcount*	*TAratio*	*TAratio*
*Industry Misvaluation*	0.6933***	0.0244	0.3341***	0.0006	0.0266***	0.0088
	(3.1742)	(0.0860)	(3.5122)	(0.0065)	(4.4150)	(0.8635)
*Long-run Performance*	0.6160***	-0.2183	0.2594***	-0.1061	0.0108**	0.0201**
	(4.0204)	(-1.0927)	(4.0373)	(-1.2469)	(2.4714)	(2.2083)
*Firm Misvaluation*	0.2840*	0.4235**	0.1601**	0.1744***	0.0030	0.0053
	(1.6960)	(2.3360)	(2.2812)	(2.6046)	(0.4622)	(1.1391)
*RD*	0.0026	-0.0351	0.0003	-0.0395***	-0.0012**	-0.0006
	(0.1246)	(-1.3909)	(0.0232)	(-3.3536)	(-2.1366)	(-0.7553)
*Size*	0.5028***	0.4421***	0.1726***	0.0879	-0.0010	0.0028
	(3.9699)	(2.7286)	(2.7637)	(1.1927)	(-0.2997)	(0.3956)
*OCF*	-0.9506	2.8558***	0.0434	0.6872	0.0350	0.0119
	(-1.1475)	(3.1077)	(0.1245)	(1.6182)	(0.8695)	(0.4525)
*Yretwd*	-0.2370**	-0.2995**	-0.1131***	-0.1255***	-0.0065*	-0.0009
	(-2.5744)	(-2.5713)	(-3.2151)	(-2.8913)	(-1.7741)	(-0.2215)
*PPE*	0.5604	-1.5443**	0.3053	-0.5119*	0.0404**	-0.0007
	(0.9353)	(-2.1400)	(0.8790)	(-1.8406)	(2.2269)	(-0.0331)
*Board*	-0.1367	0.6849	-0.1257	0.5131**	-0.0249	-0.0063
	(-0.3444)	(1.4246)	(-0.9431)	(2.0734)	(-1.6557)	(-0.2911)
*Dual*	0.2598*	0.0513	0.0802	0.0705	0.0034	0.0027
	(1.6729)	(0.3269)	(0.8138)	(1.1668)	(0.6311)	(0.3444)
*IND*	0.7358	0.3390	0.2891	0.0937	-0.0225	0.0183
	(0.5623)	(0.2479)	(0.4597)	(0.1362)	(-0.5241)	(0.3093)
*Shares Balance*	0.4160**	-0.1569	0.1691*	-0.0948	0.0061	0.0038
	(2.2568)	(-1.0295)	(1.8808)	(-1.4461)	(1.3770)	(0.8129)
*Insinvestor*	-1.2550**	-0.3559	-0.2996	0.2550	0.0152	-0.0240
	(-2.4957)	(-0.6078)	(-1.2246)	(1.1853)	(0.8821)	(-1.3628)
*Attendance*	2.0622***	1.0049	0.5998***	0.4334*	0.0336	0.0174
	(4.3085)	(1.5884)	(3.0932)	(1.6495)	(1.6604)	(0.6549)
*Board Meetings*	-0.0031	0.0239	0.0066	0.0088	0.0016***	0.0022***
	(-0.1955)	(1.4156)	(0.6943)	(1.5192)	(2.9598)	(3.2410)
*Payment*	-0.1127	-0.3836***	-0.1004**	-0.0959***	0.0181***	0.0116**
	(-1.1524)	(-2.9501)	(-2.1351)	(-2.6093)	(2.8570)	(2.3030)
*Target Type*	0.3821**	0.1922	0.2909***	0.0184	-0.0052	0.0041
	(2.3906)	(1.0441)	(4.0300)	(0.2084)	(-0.7800)	(0.6674)
Constant			-4.1980***	-2.7447	0.0482	-0.0676
			(-2.7100)	(-1.5713)	(0.5431)	(-0.4560)
Year fixed effect	Yes	Yes	Yes	Yes	Yes	Yes
Firm fixed effect	Yes	Yes	Yes	Yes	Yes	Yes
Observations	3193	2838	3855	3937	5623	5033
Pseudo R^2^	0.153	0.120	0.151	0.143		
Adj. R^2^					0.158	0.091
P-value	0.059		0.021		0.006	

Note: *TAdummy* denotes the dummy variable for the firm instigating a technological acquisition in the given year, taking a value of 1 for the occurrence of a technological acquisition and 0 otherwise. *TAcount* signifies the quantity of technological acquisitions instigated by the firm within the year. *TAratio* is a measure representing the total value of technological acquisition deals initiated by list firms during the year as a percentage of the previous year’s total assets. Within Table 7, z-values are enclosed in parentheses for columns (1) through (4), whereas t-values are reported in columns (5) and (6). Additionally, the P-values for testing the coefficient variability across groups are calculated using a permutation test bootstrap method, executed 1,000 times.

In the fixed-effects Logit regression model column (1), the coefficient for *Industry Misvaluation* is 0.6933, indicating a positive correlation with *TAdummy*. A coefficient of 0.6933 indicates that a 0.01-unit increase in *Industry Misvaluation* corresponds to approximately a 0.6957% enhancement in the odds of engaging in a technological acquisition relative to not engaging. This percentage alteration is obtained through the calculation [exp(0.6933 × 0.01) − 1] × 100%. This suggests that industry misvaluation alleviates financial constraints, thereby promoting technological acquisitions.

Despite replacing explanatory variables, the disparity in regression coefficients persists, implying that firms with high financial restraints might depend more on industry misvaluation to initiate technological acquisitions. This lends empirical support to H2. To further validate these results, we perform robustness tests using the *KZ index* [58] and the *WW index* [60]. These additional regression outcomes are detailed in S7 and S8 Tables.

## 6 Further analysis

The analysis of empirical results in this paper reveals a significant impact of industry-level overvaluation on technological acquisitions, which is primarily due to financial constraint alleviation. However, overvalued acquirers can profitably time the market and exchange equity or assets of a target company at a reduced cost, even when the synergies provided by the M&A are minimal [1]. The question then arises, in the context of the Chinese institution, whether technological acquisitions driven by industry-level stock price overvaluation lead to enhanced post-acquisition innovation outputs, rather than merely capitalizing on limited arbitrage opportunities.

Successful integration of technology following a technological acquisition is crucial for engendering innovative outputs. Nonetheless, differences in innovation practices between the acquirer and the target firm demand additional resources for adaptation and integration [64]. Firms constrained by financing may struggle to allocate adequate resources for effectively amalgamating acquired technological assets, potentially diminishing the impact of integration and the efficiency of new technology assimilation and reinvention by the acquirer. Acquirers engaging in technological acquisitions driven by industry-level overvaluation could benefit from alleviated financial constraints, potentially leading to improved efficiency in technology integration and enhanced innovation outcomes.

The regression analyses presented in Table 8 elucidate the positive influence of technological acquisition on the long-term innovation output of enterprises. Specifically, *TAdummy* variables are significantly positive at the 5% level. In columns (1) and (4), the regression coefficients for TAdummy are 0.0687 and 0.0691, respectively. Companies that engage in technological acquisitions have their *Patent* + 1 and *InvPatent* + 1 averages approximately 6.87% and 6.91% higher, respectively, than companies that do not engage in technological acquisitions. These results demonstrate that technological acquisitions not only augment the total patent output of enterprises but also significantly elevate the issuance of high-value invention patents. This underscores the pivotal role of technological acquisitions in bolstering enterprises’ innovation capabilities, particularly in fostering original and high-value invention patents.

**Table 8:** Innovation outputs.

	(1)	(2)	(3)	(4)	(5)	(6)
Variables	*Patent*	*Patent*	*Patent*	*InvPatent*	*InvPatent*	*InvPatent*
*TAdummy*	0.0687**			0.0691**		
	(2.4531)			(2.6211)		
*TAcount*		0.0282**			0.0413***	
		(2.6140)			(4.8377)	
*TAratio*			0.2894*			0.3436**
			(1.7279)			(2.2624)
Constant	4.1535***	4.1639***	4.1803***	2.3510***	2.3504***	2.3766***
	(305.1985)	(473.0251)	(945.8639)	(192.4690)	(351.3126)	(452.4728)
Year fixed effect	Yes	Yes	Yes	Yes	Yes	Yes
Firm fixed effect	Yes	Yes	Yes	Yes	Yes	Yes
Observations	6026	6026	6026	6026	6026	6026
Adj. R^2^	0.756	0.755	0.755	0.729	0.729	0.728

Note: *Patent* is the natural logarithm of the total number of patents granted by the enterprise plus one. *InvPatent* denotes the natural logarithm of the number of patents granted for the invention plus one. *TAdummy* denotes the dummy variable for the firm instigating a technological acquisition in the given year, taking a value of 1 for the occurrence of a technological acquisition and 0 otherwise. *TAcount* signifies the quantity of technological acquisitions instigated by the firm within the year. *TAratio* is a measure representing the total value of technological acquisition deals initiated by list firms during the year as a percentage of the previous year’s total assets. All t-values reported in parentheses in Table 8 use robust standard errors for clustering at the industry level. Since some of the control variables have been shown to have a significant effect on technological acquisitions, including these control variables together in the regression would likely introduce multicollinearity. Therefore, we use a regression of the three TA variables on the two measures for patents.

Furthermore, *TAcount* exhibits significant positive at both the 5% and 1% levels, indicating a positive correlation between the frequency of technological acquisitions and subsequent innovation output. This suggests that repeated technological acquisitions facilitate the accumulation of technological resources, thereby providing sustained momentum for innovation. The positive significance of *TAratio* indicates that an increase in the scale of technological acquisitions enhances their contribution to future innovation output, likely due to the acquisition of richer technological resources and greater integration potential. Hence, it is evident that technological acquisitions, motivated by industry-level overvaluation, transcend mere short-term arbitrage through share issuance for asset acquisition, and significantly contribute to the augmented innovation output of the combined entities.

## 7 Discussion

This study examines how industry-level misvaluation affects the technological acquisition decisions of Chinese listed companies and further analyzes the impact of such acquisition behavior on acquirers’ innovation output. The findings suggest that industry-level overvaluation significantly contributes to the occurrence of technological acquisitions. This result aligns with the theories of Shleifer and Vishny [1] and Rhodes-Kropf and Viswanathan [2], supporting the notion that misvaluation can drive firms’ acquisition activities.

Specifically, our study validates the role of industry-level overvaluation in facilitating technological acquisitions. Unlike previous research that primarily focused on market-level or firm-level misvaluation [3, 9], our findings address a potential research gap concerning the impact of industry-level overvaluation on acquisition decisions. Our results indicate that industry-level overvaluation exerts a more pronounced driving force on technological acquisition activities compared to firm-level overvaluation.

Additionally, the mechanism test results indicate that industry-level overvaluation significantly alleviates firms’ financial constraints, thereby facilitating technological acquisitions. This finding aligns with Gorbenko and Malenko’s [30] call for further research on the relationship between financing constraints and acquisitions, and it provides additional insights into this connection. In China, a transition economy, the unique structure of the capital market, characterized by a high proportion of retail investors, may exacerbate market volatility. This volatility, in turn, amplifies the impact of high valuations on technological acquisition decisions.

Our findings also suggest that technological acquisitions driven by excessive industry overvaluation can significantly enhance acquirers’ innovation output. This finding diverges from the perspective held by some studies, such as Zhang et al. [13], which posit that acquisitions driven by overvaluation do not generate positive synergies. Instead, our results align more closely with an alternative stream of research, exemplified by Adra and Barbopoulos [14], suggesting that firms with overvalued stocks can potentially achieve synergistic effects through acquisitions. Our study highlights the potential importance of technological acquisitions as a means to enhance firm innovation capabilities in the context of industry overvaluation.

Finally, this study not only provides a novel perspective on understanding the antecedents of technological acquisitions but also broadens the scope of open innovation research. Open innovation encompasses various approaches through which firms leverage external resources and collaborations to achieve innovation, with technological acquisitions being one such approach. Given that technological acquisitions represent a crucial pathway for implementing open innovation, our research investigates the facilitating role of industry-level overvaluation in technological acquisitions, thus elucidating the impact of external market factors on firms’ open innovation strategy choices. Additionally, this study enhances our understanding of the driving forces behind open innovation practices.

## 8 Conclusion

Our study employs a dataset of M&As by Chinese firms from 2007 to 2022 to assess the impact of industry-level overvaluation on these acquisitions and to explore the underlying mechanisms. The results indicate that such overvaluation significantly promotes technological acquisitions, a finding supported by a series of robustness tests. Further analysis indicates that industry-level overvaluation may facilitate an increase in technological acquisitions by mitigating financial constraints. Additionally, our study demonstrates that technological acquisitions, driven by industry-level overvaluation, can enhance the acquirer’s innovation output.

### 8.1 Policy implications

First, our study emphasizes the impact of industry-level overvaluation in promoting technological acquisitions, which partly elucidates the role of capital markets in supporting firms’ innovation activities. Therefore, it is crucial to optimize the capital market environment. Regulators can effectively utilize mechanisms such as margin trading and short selling to enhance market transparency, monitor abnormal trading behavior, and limit excessive speculation, thereby improving market liquidity and ensuring market stability. This will create a conducive environment for firms to pursue technological acquisitions during periods of overvaluation, thereby enhancing their innovative capabilities and long-term competitive advantage.

Second, our examination of the channeling mechanism suggests that industry-level overvaluation facilitates technological acquisition by mitigating financing constraints. This finding indicates significant financing constraints that may impede Chinese firms’ ability to undertake innovative activities. In recognition of these challenges, China has implemented several measures to improve access to capital for firms seeking to engage in innovative activities. These include establishing the Science and Technology Innovation Board and promoting reforms of the ChiNext Board and the New Third Board. To further alleviate financing constraints and support firms’ innovation-driven financing needs, we recommend that regulators continue to promote the comprehensive implementation of the registration-based IPO system. The implementation of this system streamlines the listing process, enhancing both efficiency and transparency in initial public offerings. This enables faster access to capital markets for a wider range of high-quality, technology-driven firms. This reform has the potential to address the financial needs for corporate innovation and development, thereby fostering an environment conducive to innovation-driven growth in the real economy.

### 8.2 Limitations and future research

Although this study verifies the positive impact of industry-level misvaluation on technological acquisitions and reveals the underlying channel mechanisms, several limitations exist. First, this study focuses solely on the Chinese market; future research should extend to other countries and regions to test the generalizability of the results. Future studies could investigate cross-border technological acquisitions to explore the impact of misvaluations on technological acquisitions and their innovation output in different economic contexts. This would enhance our understanding of technological acquisition on a global scale and provide broader theoretical and practical insights.

Second, while this study provides evidence that industry-level overvaluation influences technological acquisitions, future research should also examine firm- or deal-specific attributes that may be equally significant for technological acquisitions. Exploring the role and influence mechanisms of these attributes in technological acquisition will provide a more comprehensive understanding of the factors driving technological acquisition and offer firms more targeted strategic guidance in the acquisition decision-making process.

Finally, existing research has identified numerous factors affecting technological acquisition. Future studies could employ machine learning algorithms to rank the importance of these factors. Research by Zhou and Luo [65] has demonstrated the value of applying machine learning in technological acquisition to identify the most critical influencing factors. This approach can help firms optimize resource allocation in complex decision-making environments, thereby improving the success rate and subsequent innovation output of technological acquisitions.

## Supporting information

S1 Table Descriptive statistics supplement. (PDF)

S2 Table The VIF calculation results for the variables. (PDF)

S3 Table The correlation matrix with the dependent variable as TAcount. (PDF)

S4 Table The correlation matrix with the dependent variable as TAratio. (PDF)

S5 Table Results of balance tests for PSM. (PDF)

S6 Table Benchmark regression robustness tests. (PDF)

S7 Table Robustness tests for channel mechanisms using the KZ index. (PDF)

S8 Table Robustness tests for channel mechanisms using the WW index. (PDF)
