# Supplementary material for: Misvaluation and technological acquisitions: An empirical study and mechanism analysis
Source: PLoS One. 2024 Nov 14;19(11):e0313848. doi: 10.1371/journal.pone.0313848 (PMC11563430; doi:10.1371/journal.pone.0313848)
Supplement: S1 Table — (PDF) [file pone.0313848.s001.pdf]

S1 Table. Descriptive statistics supplement

Variables	Obs	Mean	Std. Dev.	Min	Median	Max	p1	p99
Net Income	11834	0.4185	3.2815	-9.5028	0.1179	150.6750	-0.8624	3.7195
Leverage	11834	0.4363	0.2131	0.0075	0.4239	0.8996	0.0556	0.8359

Note: The *Net Income* in the table is presented in billions of units.
