# Supplementary material for: Misvaluation and technological acquisitions: An empirical study and mechanism analysis
Source: PLoS One. 2024 Nov 14;19(11):e0313848. doi: 10.1371/journal.pone.0313848 (PMC11563430; doi:10.1371/journal.pone.0313848)
Supplement: S2 Table — (PDF) [file pone.0313848.s002.pdf]

S2 Table. The VIF calculation results for the variables

Variables	VIF	1/VIF
Long-run Performance	2.08	0.481723
Size	1.98	0.504777
Yretwd	1.2	0.831094
Industry Misvaluation	1.15	0.870001
OCF	1.13	0.885867
RD	1.13	0.887077
PPE	1.13	0.888814
Firm Misvaluation	1.09	0.920573
SA	1.08	0.924164
Board	1.07	0.933639
Dual	1.08	0.926743
IND	1.23	0.812112
Shares Balance	1.08	0.923039
Insinvestor	1.39	0.716989
Attendance	1.12	0.889820
Board Meetings	1.12	0.896582
Payment	1.51	0.663459
Target Type	1.60	0.625477
Mean VIF	1.34	
