# Supplementary material for: Misvaluation and technological acquisitions: An empirical study and mechanism analysis
Source: PLoS One. 2024 Nov 14;19(11):e0313848. doi: 10.1371/journal.pone.0313848 (PMC11563430; doi:10.1371/journal.pone.0313848)
Supplement: S3 Table — (PDF) [file pone.0313848.s003.pdf]

S3 Table. The correlation matrix with the dependent variable as TAcount

	TAcount	*Industry Misvaluation*	*Long-run Performance*	*Firm Misvaluation*	*RD*	*Size*	*OCF*	*Yretwd*	*PPE*	*Board*	*Dual*	*IND*	*Shares Balance*	*Insinvestor*	*Attendance*	*Board Meetings*	*Payment*	*Target Type*
TAcount	1																	
Industry Misvaluation	0.086***	1																
Long-run Performance	0.062***	0.022**	1															
Firm Misvaluation	0.015*	0.00600	0.00100	1														
RD	0.126***	0.041***	0.267***	0.115***	1													
Size	0.052***	0.082***	-0.671***	0.039***	-0.164***	1												
OCF	0.018**	0.030***	0.118***	0.047***	-0.00700	0.066***	1											
Yretwd	-0.066***	0.313***	0.024***	0.226***	0.00200	-0.00600	0.079***	1										
PPE	-0.063***	-0.037***	-0.129***	-0.039***	-0.197***	0.083***	0.220***	0.046***	1									
Board	-0.067***	-0.059***	-0.193***	-0.0100	-0.093***	0.206***	0.027***	0.030***	0.106***	1								
Dual	0.074***	0.031***	0.155***	0.067***	0.105***	-0.146***	-0.00900	-0.017*	-0.101***	-0.147***	1							
IND	0.019**	0.031***	0.035***	0.017*	0.051***	-0.024***	-0.00300	-0.027***	-0.045***	-0.424***	0.103***	1						
Shares Balance	0.039***	0.019**	0.120***	0.046***	0.143***	-0.088***	-0.025***	-0.023***	-0.090***	0.00100	0.042***	0	1					
Insinvestor	-0.087***	-0.028***	-0.249***	0.117***	-0.154***	0.369***	0.122***	0.066***	0.125***	0.214***	-0.161***	-0.084***	-0.216***	1				
Attendance	0.00900	-0.00300	0.148***	0.074***	-0.0140	-0.058***	0.106***	-0.00600	-0.065***	-0.00900	0.070***	0.00600	-0.0100	0.201***	1			
Board Meetings	0.029***	0.082***	-0.174***	0.070***	-0.032***	0.245***	-0.097***	0.069***	-0.048***	-0.040***	-0.017**	0.046***	0.00200	0.030***	-0.103***	1		
Payment	0.077***	0.093***	0.027***	0.0110	0.060***	0.046***	0.00400	-0.072***	-0.039***	-0.095***	0.073***	0.056***	0.058***	-0.090***	-0.00700	0.054***	1	
TargetType	0.146***	0.116***	0.026***	0.016*	0.083***	0.071***	0.032***	-0.132***	-0.061***	-0.134***	0.088***	0.067***	0.102***	-0.128***	-0.030***	0.019**	0.577***	1
