# Supplementary material for: Misvaluation and technological acquisitions: An empirical study and mechanism analysis
Source: PLoS One. 2024 Nov 14;19(11):e0313848. doi: 10.1371/journal.pone.0313848 (PMC11563430; doi:10.1371/journal.pone.0313848)
Supplement: S5 Table — (PDF) [file pone.0313848.s005.pdf]

S5 Table. Results of Balance Tests for PSM

Variable	Sample	Treated	Control	%bias	%reduct bias	t	t-test p>t
Topholder	Unmatched	33.125	32.345	5.5		3.01	0.003
	Matched	32.2	32.332	-0.9	83.1	-1	0.317
Cashdebtratio	Unmatched	0.19771	0.18067	4.2		2.26	0.024
	Matched	0.17448	0.18207	-1.9	55.4	-1.47	0.143
Intangibles	Unmatched	0.11151	0.0469	12.5		6.26	0
	Matched	0.04792	0.04874	-0.2	98.7	-0.79	0.431
SA	Unmatched	-3.7836	-3.7985	5.9		3.23	0.001
	Matched	-3.8034	-3.8059	1	83.6	-0.32	0.748
Caprdprofit	Unmatched	14.65	10.767	6.6		3.47	0.001
	Matched	10.14	11.402	-2.2	67.5	-1.51	0.131

Note: Our covariates include *Cashdebtratio* (net cash flow from operating activities / total liabilities) as an important indicator of financial health, reflecting the firm's debt repayment ability and liquidity. *Topholder* (proportion of shares held by the largest shareholder) measures the company's governance structure, controlling for differences in governance levels and avoiding confounding effects of governance structure on technological acquisition behavior. *Caprdprofit* (capitalized R&D expenditure as a proportion of current net profit) indicates R&D investment and innovation capability. *SA*(degree of financial constraints of the acquirer), and *Intangibles* (increase in intangible assets) reflect the firm's accumulation and outcomes in technology and innovation. These covariates ensure that the matched treatment and control groups do not exhibit significant differences in key aspects of financial health, governance, R&D investment, and innovation outcomes, supporting further model estimation.
