# Supplementary material for: Misvaluation and technological acquisitions: An empirical study and mechanism analysis
Source: PLoS One. 2024 Nov 14;19(11):e0313848. doi: 10.1371/journal.pone.0313848 (PMC11563430; doi:10.1371/journal.pone.0313848)
Supplement: S6 Table — (PDF) [file pone.0313848.s006.pdf]

S6 Table. Benchmark Regression Robustness Tests

	Panel A			Panel B		
	(1)	(2)	(3)	(1)	(2)	(3)
	Logit	Poisson	OLS	Logit	Poisson	OLS
Variables	*TAdummy*	*TAcount*	*TAratio*	*TAdummy*	*TAcount*	*TAratio*
Industry Misvaluation	0.2579***	0.1339**	0.0405***	0.5587***	0.2468***	0.0155**
	(3.4332)	(2.0585)	(4.3053)	(3.4654)	(2.8470)	(2.5532)
Long-run Performance	0.2860***	0.2271***	0.0509***	0.4451***	0.1957***	0.0174***
	(6.0023)	(5.5238)	(8.5522)	(3.7475)	(3.1789)	(3.3365)
Firm Misvaluation	0.0732	0.0426	0.0176***	0.1512	0.0819	0.0020
	(1.6436)	(1.1787)	(3.3443)	(1.2642)	(1.3578)	(0.6127)
RD	0.0148***	0.0074**	0.0010*	0.0059	-0.0086	-0.0004
	(3.0150)	(1.9762)	(1.7408)	(0.3890)	(-0.8265)	(-1.0558)
Size	0.3310***	0.2291***	0.0137***	0.5738***	0.1922***	0.0029
	(13.5559)	(11.4853)	(4.6823)	(6.2159)	(6.0944)	(0.9248)
OCF	0.2726	0.2844	0.0247	1.1308*	0.6453**	0.0269
	(1.0330)	(1.2441)	(0.7607)	(1.8238)	(2.1558)	(1.2097)
Yretwd	-0.1222***	-0.0834***	-0.0077*	-0.2750***	-0.1267***	-0.0031
	(-3.7585)	(-2.9068)	(-1.9101)	(-3.7081)	(-4.8098)	(-1.2631)
PPE	0.1020	-0.0662	0.0271	0.1820	0.1758	0.0236*
	(0.7118)	(-0.5403)	(1.5553)	(0.4225)	(0.5420)	(1.7380)
Board	-0.2051**	-0.1053	-0.0256**	0.1177	0.2058	-0.0224
	(-2.0125)	(-1.2511)	(-2.1142)	(0.4067)	(1.5850)	(-1.6387)
Dual	0.0519	0.0742**	0.0083*	-0.0034	-0.0003	0.0031
	(1.3903)	(2.5237)	(1.9193)	(-0.0320)	(-0.0063)	(0.8059)
IND	-0.8339**	-0.6259**	-0.0458	-0.5744	-0.0635	-0.0245
	(-2.5164)	(-2.2488)	(-1.1526)	(-0.6101)	(-0.2034)	(-0.6417)
Shares Balance	-0.0282	-0.0300	0.0005	-0.1162	-0.0735	0.0023
	(-0.9837)	(-1.3372)	(0.1546)	(-1.0906)	(-1.3034)	(0.5635)
Insinvestor	-0.3424***	-0.2368***	-0.0494***	-0.5268	0.1161	-0.0044
	(-3.9707)	(-3.4594)	(-4.9798)	(-1.4735)	(0.8765)	(-0.4302)
Attendance	0.4531***	0.2524**	0.0503***	1.5484***	0.5029***	0.0233*
	(3.6618)	(2.5041)	(3.4704)	(4.4633)	(3.9136)	(1.8287)
Board Meetings	0.0044	0.0067*	0.0038***	-0.0037	0.0050	0.0017***
	(1.0027)	(1.8769)	(7.2246)	(-0.3175)	(1.2472)	(3.3787)
Payment	-0.0798**	-0.0697**	0.0103**	-0.2629***	-0.1132**	0.0118***
	(-2.2148)	(-2.1307)	(2.2540)	(-3.2834)	(-2.3717)	(2.9240)
Target Type	0.1427***	0.1583***	0.0150**	0.2557**	0.1295*	-0.0022
	(2.6435)	(3.1105)	(2.1383)	(2.1105)	(1.7346)	(-0.6300)
Constant	-9.6798***	-7.2322***	-0.7082***		-4.8376***	-0.0262
	(-15.0449)	(-11.9190)	(-8.9847)		(-5.7674)	(-0.3013)
Observations	11834	11834	11834	5848	7085	9400
Adj. R^2						0.128
Pseudo R^2				0.154	0.149	

Note: *TAdummy* denotes the dummy variable for the firm instigating a technological acquisition in the given year, taking a value of 1 for the occurrence of a technological acquisition and 0 otherwise. *TAcount* signifies the quantity of technological acquisitions instigated by the firm within the year. *TAratio* is a measure representing the total value of technological acquisition deals initiated by list firms during the year as a percentage of the previous year's total assets. The Probit model is used to assess the binary variable (*TAdummy*), due to its ability to handle binary outcomes. However, as the Probit model cannot accommodate individual fixed effects, we include additional dummy variables for the year and industry. For *TAcount*, which measures the count of technological acquisitions, the negative binomial model is appropriate, given its utility with discrete count data. *TAratio*, which is a proportion between 0 and 1, is modeled using the Tobit approach, suitable for dependent variables with bounded values. Here, we also introduce dummy variables for year and industry. The Panel A regression results indicate that the research conclusions of this study remain consistent, regardless of alterations to model settings. Furthermore, this paper performs a robust test by excluding years marked by major market events—the 2008 financial crisis, the 2015 stock market crash, and the 2020 COVID-19 impact. Panel B regression, employing Logit, Poisson, and OLS models with controls for year and firm fixed effects and standard errors clustered at the industry level, reaffirms the robustness of the benchmark analysis, even when excluding these tumultuous periods.
