# Supplementary material for: Misvaluation and technological acquisitions: An empirical study and mechanism analysis
Source: PLoS One. 2024 Nov 14;19(11):e0313848. doi: 10.1371/journal.pone.0313848 (PMC11563430; doi:10.1371/journal.pone.0313848)
Supplement: S7 Table — (PDF) [file pone.0313848.s007.pdf]

S7 Table. Robustness Tests for Channel Mechanisms Using the KZ Index

	(1)	(2)	(3)	(4)	(5)	(6)
	High FC	*Low FC*	*High FC*	*Low FC*	*High FC*	*Low FC*
	Logit		Poisson		OLS	
Variables	*TAdummy*	*TAdummy*	*TAcount*	*TAcount*	*TAratio*	*TAratio*
Industry Misvaluation	0.8170***	-0.0640	0.2457**	0.1620	0.0262***	0.0105
	(3.1370)	(-0.2547)	(2.0171)	(1.2508)	(4.0960)	(1.0358)
Long-run Performance	0.3301*	0.0196	0.0933	0.0401	0.0181***	0.0105
	(1.9069)	(0.1080)	(1.0533)	(0.5630)	(3.8406)	(1.4520)
Firm Misvaluation	0.3979**	-0.1178	0.1524**	0.0657	0.0086*	-0.0008
	(2.1924)	(-0.7281)	(2.0902)	(0.8649)	(1.9487)	(-0.1587)
RD	-0.0192	-0.0094	-0.0265*	-0.0183**	-0.0011	-0.0008
	(-0.7898)	(-0.4713)	(-1.9037)	(-2.3442)	(-1.1788)	(-0.9041)
Size	0.5227***	0.5060***	0.1882***	0.2310***	0.0010	0.0040
	(3.4750)	(4.0379)	(3.0039)	(3.2009)	(0.2828)	(0.8404)
OCF	0.6510	0.6344	0.2697	0.2476	0.0062	0.0458*
	(0.7034)	(0.7739)	(0.7213)	(0.5621)	(0.1945)	(1.7750)
Yretwd	-0.3031***	-0.2206**	-0.1380***	-0.0694	-0.0035	-0.0034
	(-2.9407)	(-2.1964)	(-2.5770)	(-1.2994)	(-0.9794)	(-0.9968)
PPE	1.3676**	-0.8568	0.7240	-0.5047	0.0422*	0.0059
	(2.0048)	(-1.4438)	(1.5316)	(-1.2846)	(1.7932)	(0.2475)
Board	-0.0412	0.4674	-0.2249	0.5137***	-0.0282*	0.0015
	(-0.0946)	(1.1740)	(-0.8809)	(3.0127)	(-1.9062)	(0.1012)
Dual	-0.0699	-0.0293	0.0047	-0.0185	0.0016	-0.0015
	(-0.4188)	(-0.2034)	(0.0677)	(-0.2843)	(0.3016)	(-0.2322)
IND	-0.4090	-0.0514	-0.1947	0.2546	0.0019	-0.0431
	(-0.2317)	(-0.0359)	(-0.2524)	(0.3166)	(0.0205)	(-0.7138)
Shares Balance	0.0473	0.0674	-0.0081	-0.1302	0.0011	0.0070
	(0.2264)	(0.3909)	(-0.1083)	(-1.3963)	(0.1702)	(1.1371)
Insinvestor	-1.6837**	-0.4260	-0.4806	0.7453**	-0.0272	-0.0157
	(-2.1557)	(-0.5597)	(-1.3110)	(1.9805)	(-1.0602)	(-0.7704)
Attendance	2.1643***	2.7805***	0.7493**	0.8766**	0.0571*	0.0090
	(2.8851)	(3.2577)	(2.0818)	(2.3366)	(1.9848)	(0.3494)
Board Meetings	-0.0001	-0.0298	-0.0027	-0.0116	0.0019**	0.0019*
	(-0.0043)	(-1.2631)	(-0.4916)	(-1.1103)	(2.2365)	(1.9825)
Payment	-0.1609	-0.1719	-0.0424	-0.0371	0.0174***	0.0070
	(-1.4193)	(-1.5462)	(-0.8314)	(-0.6883)	(2.8064)	(1.1702)
Target Type	0.2303	0.2028	0.0677	0.2119**	0.0021	-0.0031
	(1.2862)	(1.2227)	(1.0376)	(2.4525)	(0.6552)	(-0.3968)
Constant			-3.5932**	-6.5564***	-0.0003	-0.0728
			(-1.9935)	(-3.9559)	(-0.0029)	(-0.7479)
Year fixed effect	Yes	Yes	Yes	Yes	Yes	Yes
Firm fixed effect	Yes	Yes	Yes	Yes	Yes	Yes
Observations	2758	3202	3625	4044	5234	5282
Pseudo R^2	0.177	0.124	0.148	0.149		
Adj. R^2					0.171	0.097
P-value	0.0125		0.380		0.016	

Note: *TAdummy* denotes the dummy variable for the firm instigating a technological acquisition in the given year, taking a value of 1 for the occurrence of a technological acquisition and 0 otherwise. *TAcount* signifies the quantity of technological acquisitions instigated by the firm within the year. *TAratio* is a measure representing the total value of technological acquisition deals initiated by list firms during the year as a percentage of the previous year's total assets. Within Table, z-values are enclosed in parentheses for columns (1) through (4), whereas t-values are reported in columns (5) and (6). Additionally, the P-values for testing the coefficient variability across groups are calculated using a permutation test bootstrap method, executed 1,000 times.
