# Supplementary material for: Misvaluation and technological acquisitions: An empirical study and mechanism analysis
Source: PLoS One. 2024 Nov 14;19(11):e0313848. doi: 10.1371/journal.pone.0313848 (PMC11563430; doi:10.1371/journal.pone.0313848)
Supplement: S8 Table — (PDF) [file pone.0313848.s008.pdf]

S8 Table. Robustness Tests for Channel Mechanisms Using the WW Index

	(1)	(2)	(3)	(4)	(5)	(6)
	High FC	*Low FC*	*High FC*	*Low FC*	*High FC*	*Low FC*
	Logit		Poisson		OLS	
Variables	*TAdummy*	*TAdummy*	*TAcount*	*TAcount*	*TAratio*	*TAratio*
Industry Misvaluation	0.7562***	-0.1058	0.4832***	-0.1898	0.0197***	0.0165
	(2.9684)	(-0.4113)	(5.0809)	(-1.5440)	(3.2727)	(1.5553)
Long-run Performance	0.6505***	-0.1078	0.2473***	-0.0710	0.0225***	-0.0005
	(3.7556)	(-0.5629)	(3.3861)	(-0.7389)	(3.2017)	(-0.0514)
Firm Misvaluation	0.4979***	-0.1198	0.2211***	-0.0530	0.0043	-0.0011
	(3.0347)	(-0.6308)	(3.2240)	(-0.4239)	(0.9268)	(-0.1298)
RD	0.0064	-0.0110	-0.0115	-0.0117	-0.0010	-0.0014*
	(0.2603)	(-0.4917)	(-1.1112)	(-0.9408)	(-1.0679)	(-1.9225)
Size	0.7783***	0.1264	0.1686***	0.1251	0.0085**	-0.0078
	(5.4372)	(0.9144)	(3.3753)	(1.4621)	(2.3983)	(-1.4046)
OCF	-0.5797	0.9094	-0.0493	0.6244	-0.0143	0.0430
	(-0.6362)	(0.9987)	(-0.1171)	(1.1090)	(-0.5334)	(1.3150)
Yretwd	-0.2420**	-0.1426	-0.1396***	-0.0640	-0.0013	-0.0009
	(-2.3665)	(-1.3482)	(-3.6344)	(-1.6000)	(-0.3864)	(-0.2197)
PPE	2.2909***	-0.8239	0.9405**	-0.4031	0.0574**	-0.0192
	(3.4041)	(-1.3349)	(2.2706)	(-1.0588)	(2.5972)	(-0.8320)
Board	-0.4468	0.8487*	-0.0645	0.4166***	-0.0230*	0.0159
	(-1.1014)	(1.8444)	(-0.2978)	(2.6468)	(-1.8347)	(0.7755)
Dual	-0.0869	0.2924*	-0.0218	0.1261*	0.0035	0.0019
	(-0.5718)	(1.7876)	(-0.3877)	(1.6736)	(0.7388)	(0.2676)
IND	1.5877	-2.4152*	-0.1207	-0.7129	-0.0280	-0.0409
	(1.2098)	(-1.7642)	(-0.1867)	(-0.9049)	(-0.6426)	(-0.7367)
Shares Balance	-0.2196	0.4555***	-0.1339**	0.0464	0.0050	0.0100*
	(-1.3428)	(2.6616)	(-1.9769)	(0.5946)	(0.7944)	(1.8766)
Insinvestor	-0.6245	-0.5452	-0.2063	-0.0398	-0.0124	0.0243
	(-1.1193)	(-1.0354)	(-1.0796)	(-0.1410)	(-0.7907)	(1.3229)
Attendance	1.4159***	1.8395***	0.2508	0.8974***	0.0137	0.0419*
	(2.8486)	(3.3432)	(1.1596)	(3.9299)	(1.0415)	(1.6984)
Board Meetings	-0.0078	-0.0013	0.0057	-0.0047	0.0020***	0.0016*
	(-0.4897)	(-0.0757)	(1.4640)	(-0.5286)	(4.0941)	(1.8924)
Payment	-0.2893**	-0.2053*	-0.1222***	-0.0253	0.0090	0.0156***
	(-2.5580)	(-1.7696)	(-2.6168)	(-0.3686)	(1.5774)	(3.1214)
Target Type	0.4755***	0.1059	0.1845**	0.0843	0.0020	-0.0054
	(2.8794)	(0.5699)	(2.2090)	(0.9226)	(0.5295)	(-0.6264)
Constant			-3.5820**	-3.3858*	-0.1544*	0.1310
			(-2.3724)	(-1.7590)	(-1.9584)	(0.9574)
Year fixed effect	Yes	Yes	Yes	Yes	Yes	Yes
Firm fixed effect	Yes	Yes	Yes	Yes	Yes	Yes
Observations	3401	2543	4506	3101	6157	4265
Pseudo R^2	0.200	0.153	0.150	0.160		
Adj. R^2					0.137	0.102
P-value	0.018		0.000		0.000	

Note: *TAdummy* denotes the dummy variable for the firm instigating a technological acquisition in the given year, taking a value of 1 for the occurrence of a technological acquisition and 0 otherwise. *TAcount* signifies the quantity of technological acquisitions instigated by the firm within the year. *TAratio* is a measure representing the total value of technological acquisition deals initiated by list firms during the year as a percentage of the previous year's total assets. Within Table, z-values are enclosed in parentheses for columns (1) through (4), whereas t-values are reported in columns (5) and (6). Additionally, the P-values for testing the coefficient variability across groups are calculated using a permutation test bootstrap method, executed 1,000 times.
